# A review of carbon-based and non-carbon-based catalyst supports for the selective catalytic reduction of nitric oxide

**DOI:** 10.3762/bjnano.9.68

**Published:** 2018-02-27

**Authors:** Shahreen Binti Izwan Anthonysamy, Syahidah Binti Afandi, Mehrnoush Khavarian, Abdul Rahman Bin Mohamed

**Affiliations:** 1 School of Chemical Engineering, Universiti Sains Malaysia, Engineering Campus, 14300 Nibong Tebal, Penang, Malaysia

**Keywords:** carbon-based catalyst support, Eley–Riedeal mechanism, Langmuir–Hinshelwood mechanism, nitric oxide, non-carbon-based catalyst support, selective catalytic reduction

## Abstract

Various types of carbon-based and non-carbon-based catalyst supports for nitric oxide (NO) removal through selective catalytic reduction (SCR) with ammonia are examined in this review. A number of carbon-based materials, such as carbon nanotubes (CNTs), activated carbon (AC), and graphene (GR) and non-carbon-based materials, such as Zeolite Socony Mobil–5 (ZSM-5), TiO_2_, and Al_2_O_3_ supported materials, were identified as the most up-to-date and recently used catalysts for the removal of NO gas. The main focus of this review is the study of catalyst preparation methods, as this is highly correlated to the behaviour of NO removal. The general mechanisms involved in the system, the Langmuir–Hinshelwood or Eley–Riedeal mechanism, are also discussed. Characterisation analysis affecting the surface and chemical structure of the catalyst is also detailed in this work. Finally, a few major conclusions are drawn and future directions for work on the advancement of the SCR-NH_3_ catalyst are suggested.

## Review

### Introduction

Nowadays, air pollution has become a major environmental problem, which affects the natural characteristics of the atmosphere (gaseous constituents, water vapour that determines humidity and ozone layer) and adversely impacts the environment and human health. Nitrogen oxides (NO*_x_*, *x* = 1, 2) are irritant gases that cause problems such as photochemical smog, formation of fine particles (PM), acid rain, ozone depletion, and the release of greenhouse gases to the environment [1]. NO is considered to be the most difficult gas to remove from the atmosphere because it behaves like a supercritical fluid at room temperature. On the other hand, NO_2_ can easily be removed because of its propensity to dissolve in water. Thus, the difficulty in NO elimination is the main challenge in NO*_x_* gas removal. In fact, NO is the main pollutant, representing about 90% of total NO*_x_* [2]. The main source of NO*_x_* emissions is dominated by stationary sources, which originate from industrial processes, energy production and distribution, transportation, and agriculture. In large cities where motor vehicle traffic is high, a huge amount of NO*_x_* is released into the environment. This phenomenon can significantly cause detriment to public health. To overcome this problem, maximum standards for NO*_x_* emissions have been reinforced in the transportation sector. These guidelines and regulations must be adhered to and numerous efforts taken in order to reduce NO*_x_* emissions in the atmosphere. However, more beneficial and economic ways to remove NO are still lacking and are yet to be practically evaluated.

In recent years, many abatement technologies including dry and wet techniques have been utilised in industrial boilers and power plants to control NO*_x_* emissions. Post-combustion such as selective catalytic reduction (SCR), non-selective catalytic reduction (NSCR), adsorption, corona discharge, electrochemical cell, radiation, and the wet system are examples of the dry technique while the scrubber is an example of the wet technique [3]. Of these technologies, due to its effectiveness, low cost, and high selectivity, the SCR of NO has emerged as the most promising method and has been widely applied over several decades to meet the stringent regulation of NO emissions from stationary sources [4]. Meanwhile, the three-way catalyst with carbon monoxide (CO) and hydrocarbon is also used in mobile sources to remove NO from gasoline, but this technology is limited to diesel and lean-burn gasoline engines. In the SCR of NO, a reducing agent must be introduced into the system in order to successfully convert NO into nitrogen (N_2_), inert gases such as ammonia (NH_3_), and urea [5]. The reaction system reduces the Gibbs free energy values initiated by the reducing agent; the introduction of oxygen also contributes to this occurrence. According to results from the literature, NH_3_ is the most practical reducing agent in the SCR of NO, as it results in high NO removal. The catalyst plays an important role in enhancing NO-SCR technology. The most widely used catalyst is V_2_O_5_–WO_3_/TiO_2_ due to its excellent catalytic activity and its high resistance to sulphur poisoning [6]. However, some problems are still evident with the use of this catalyst, e.g., the vanadium species is highly toxic to humans and the environment, and excessive dust pollution will usually result from the upstream flue gas, which can cause the deactivation of the catalyst. In addition, the catalyst also has a limited temperature window (300–400 °C) and poor thermal stability [7–8]. Hence, there is an urgent need to find other catalysts that can overcome these drawbacks and also have high resistance to water (H_2_O) and oxidation of SO_2_ to SO_3_, so that high activity and selectivity, high thermal stability, and a temperature lower than 300 °C can be achieved, thus increasing the lifetime of the catalyst. Lower-temperature SCR is foremost in importance to ensure the complete removal of NO. This is because of the wide temperature window of 150–500 °C in a diesel engine as well as the low energy consumption and economy of NH_3_-SCR [9].

In the literature, abundant catalysts at low temperature have been explored such as transitional metals (Mn, Cu, Ce, Fe, Co, Mo) [10–13], novel metals (Pt, Pd) [14], and metal ion-exchange zeolite catalysts [15]. The Mn-based catalyst is one of the most active metal oxide catalysts for high N_2_ selectivity at low temperature and is recognised by many researchers as a potential alternative for the common catalyst. Jin et al. [16] successfully used a Mn-based catalyst with the addition of Ce to improve the activity and stability of SCR performance. It is well known that Ce is a good additive that can provide enough oxygen groups with the ability to release or store oxygen, thus improving the catalytic activity of SCR. In addition, over the last few years, the use of carbon-based catalysts have been widely studied due to their high surface area, porosity, ability to regenerate and be reused, and good support properties [17]. Several metal oxides were impregnated with carbon-based materials such as carbon nanotubes (CNTs), activated carbon (AC), activated carbon nanofibres (ACNFs), and graphene (GR). It is highly favourable to use these carbon-based materials as a catalyst support in the SCR system, as they can provide sites for active metals and protect the metals from sintering. However, they are also significantly affected by H_2_O and SO_2_ poisoning.

There are various methods for preparing a catalyst such as impregnation, sol–gel, incipient-wetness, co-precipitation, electroplating, and the polyol process. Some of the catalysts prepared via these methods can achieve low temperatures of SCR with content resistance of H_2_O and SO_2_. Each method is different from the other, and thus each affects the structure and physical and chemical properties of the catalyst. In fact, choosing the best method for catalyst preparation is crucial in order to increase the interaction between the metal and support, and to obtain a desirable catalyst particle size. Lázaro et al. [17] summarised the carbon-based catalysts for SCR at low temperature and compared it with commercial catalysts. A good example can be seen in Chuang et al. [18], where a comparative study was made between the preparation of AC-supported Cu catalysts through impregnation, the polyol process, and microwave-heated polyol process using Cu(NO_3_)·3H_2_O as the copper precursor.

To date, many researchers have reviewed the SCR of NO at low temperature. Li et al. [9] reviewed low-temperature SCR on metal oxide and zeolite catalysts with a focus on catalyst performance and taking into account other possible mechanisms. Meanwhile, Mrad et al. [5] reviewed and summarised the use of a hydrocarbon catalyst in the SCR of NO. However, not much effort has been made to summarise the progress of catalyst synthesis methods to achieve catalytic activity at low-temperature SCR. Therefore, this paper addresses this gap by reviewing the development of catalyst preparation methods.

In this paper, the current progress in research on low-temperature SCR of NO over metal and bi-metallic oxides with carbon-based catalysts is reviewed. Furthermore, this review provides a comprehensive focus on the methods of catalyst preparation that can provide high activity, high stability, and convey good resistance towards H_2_O and SO_2_. The reaction mechanism for NO removal is also discussed. Finally, major conclusions and future directions for research on the SCR of NO are presented.

### Mechanism of NO removal

The adsorption behaviour of gas on the catalyst surface is known to be an important factor for understanding the chemical nature of SCR. In NH_3_-SCR, NO is targeted and reduced to N_2_, which can then be safely released into the surroundings. The typical mechanism for the SCR-NH_3_ of NO, which involves O_2_, is outlined in Equation 1 [19].

[1]
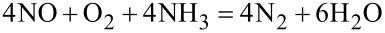


In the absence of O_2_, the reaction mechanism follows Equation 2.

[2]
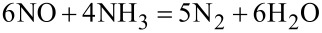


Equation 1 is recommended as the standard reaction for NH_3_-SCR of NO, as more than 90% of NO is present among the NO*_x_* species and thus controlling the reaction stoichiometry. In the standard SCR mechanism of Equation 1, one molecule of NH_3_ is required to reduce one molecule of NO with the excess O_2_.

However, fast SCR may also occur in the presence of O_2_ with NO and NO_2_ over active components. In the presence of O_2_, NO_2_ can be formed when NO is being oxidised by active oxygen. The general reactions are described by Equation 3 and Equation 4 [20].

[3]
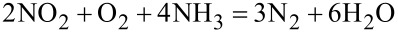


[4]
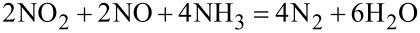


Fast SCR and standard SCR can be distinguished according to the formation of NO_2_. In fast SCR, nitrous acid (HNO_2_) and nitric acid (HNO_3_) are formed from the dimerisation of NO_2_ [2]. Then, an ammonium nitrate (H_4_NNO_3_) intermediate is formed when NH_3_ reacts with HNO_3_ and subsequently decomposes to N_2_O and H_2_O by NO at higher temperature [20].

It is noted that NO_2_ is highly crucial in the fast SCR reaction, as NO_2_ is a more powerful oxidising agent compared to O_2_. Thus, the reaction between NO and HNO_3_ is significant in determining the rate-limiting step of fast SCR. According to Nova et al. [20], a ratio of NO/NH_3_ approaching 1 is good for increasing the reaction rate, whereby the formation of NO_2_ will be inhibited when the NH_3_ concentration is increased, which restrains the formation of HNO_3_. Indeed, Nova et al. [20] also addressed the mechanism of fast SCR reaction over V_2_O_5_–WO_3_/TiO_2_ catalysts. They concluded that NO_2_ is vital for attaining faster reoxidation of the vanadium sites.

There are two surface reactions that are usually involved in the NO removal system: the Langmuir–Hinshelwood and Eley–Rideal mechanisms [9]. It is believed that NH_3_ is first adsorbed by both Lewis acid sites and Brønsted acid sites followed by NO and O_2_.

#### Langmuir–Hinshelwood mechanism

The Langmuir–Hinshelwood mechanism occurs when two molecules of NO and NH_3_ are adsorbed to the catalyst surface and then bonded together to form N_2_ and H_2_O. Liu et al. [21] examined the Langmuir–Hinshelwood mechanism over a MnO*_x_*-CeO_2_ catalyst reaction. The adsorbed NH_3_ on the Lewis acid sites formed activated NH_3_, which is a NH_2_^−^ species, in the oxidation dehydrogenation process. The NO that was oxidised to NO_2_ on the catalyst surface reacted with NH_2_^−^ to produce intermediate nitrosamine (NH_2_NO). The proposed Langmuir–Hinshelwood reaction scheme is outlined by Equation 5–9.

[5]
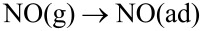


[6]
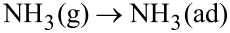


[7]
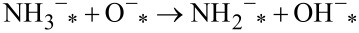


[8]
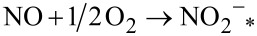


[9]
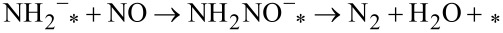


where _*_ represents the Lewis acid sites on the catalyst surface.

Firstly, gaseous NO and NH_3_ are adsorbed on the catalyst surface, as per Equation 5 and Equation 6. As the reaction starts with NH_3_, NH_3_ will be adsorbed on the Lewis acid sites and Brønsted acid sites and then oxidised into coordinated NH_3_ species and ionic NH_4_^+^, while NO is oxidised into NO_2_ via Equation 7 and Equation 8, respectively. The manganese and ceria cations used in this reaction contribute to the large amount of Lewis acid sites. It is noted that the NH_4_^+^ formed on the Brønsted acid sites are not necessary in the SCR reaction at low temperature. Furthermore, the oxidised NO reacts with adjacent activated NH_3_ species to form intermediate NH_2_NO^−^. After that, it will decompose into N_2_ and H_2_O, as per Equation 9.

#### Eley–Rideal mechanism

The Eley–Rideal mechanism suggests that only one molecule will adsorb on the catalyst surface and the second molecule will react with it directly from the gas phase (van der Waals force) to form a new product. Liu et al. [22] found that the Fe_2_O_3_ catalyst forms amide species (NH_2_^−^) in the presence of NH_3_ on the Lewis acid sites and reacted directly with gaseous NO to form N_2_ and H_2_O, as per Equation 10–12, respectively:

[10]
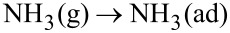


[11]
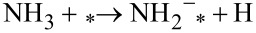


[12]
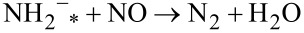


In addition, Cha et al. [19] also proposed the Eley–Rideal mechanism involving CeO_2_. The higher quantity of Ce^3+^ ions over the Ce^4+^ ions facilitated the oxidation of NH_3_ to N_2_ and H_2_O for NO reduction in the SCR reaction. High Ce^3+^ ion concentration contributed to the high amount of chemisorbed oxygen, which increased the redox cycle and enhanced the reducibility of hydrogen. This is because the Ce^3+^ ions will assist the migration of O_2_ from gas phase to the surface of the catalyst [8,23]. Afterwards, Wei et al. [24] explained that NO*_x_* removal using FeCu/zeolite catalyst followed both Eley–Rideal and Langmuir–Hinshelwood mechanisms. First, NO*_x_* directly reacted with oxidised NH_3_ to express the Eley–Rideal mechanism, and the NO species reacted with NH_3_ species adsorbed to the Fe^2+^ and Cu^2+^ ions, following the Langmuir–Hinshelwood mechanism. Shi et al. [25] used DRIFT to investigate the chemical reaction of reactants with the Lewis acid site and Brønsted acid site to improve activity at low temperature. Chen et al. [26] found that CeO_2_ provided both acid sites, while WO_3_ helped in increasing the Brønsted acid sites in the CeO_2_-WO_3_ mixed oxide catalyst.

Generally, chemical reactions that occurr in the NO adsorption system involve a redox reaction on the surface of the catalyst and adsorption of NO, NH_3_, and O_2_. The rate of a chemical reaction normally depends on the catalyst, which speeds up the reaction with less energy being required. Thus, the addition of a support for the catalyst will only increase the rate of reaction for NO removal. A redox reaction is a chemical reaction that involves the simultaneous occurrence of the oxidation and reduction process. This reaction occurs during the preparation of the catalyst between the active components and selected support. According to Liu et al. [27], the electronic inductive interaction between Fe^3+^ (metal) and Ti^4+^ (support) species will increase the oxidation ability of Fe^3+^ to Fe^2+^ species, which will then lead to the enhancement of surface active sites to readily react with NO. The synergetic effect of the metal cations present is important to increase the catalytic activity in the SCR of NO.

### Catalyst preparation

The success of nitric oxide removal in a SCR reaction depends entirely on catalyst stability and activity [28]. The synthesis method is one of the key factors that contribute towards an even distribution of the active component and good particle properties [29]. Different preparation methods have been carried out by modifying the current SCR catalyst to achieve high NO selectivity in the NH_3_-SCR system. Impregnation is one of the most commonly used techniques due to its simplicity [30]. This method starts with the inclusion of metals (Zn, Cu, Ni, Co, Fe or Mn) into the catalyst support material. A metal solution precursor is commonly added together with the support material. After mixing, the surface material is allowed to fully submerge with the selection of metals through continuous mixing. Sonication or stirring methods are applied to assure the upmost dispersion of metal in the solution precursor [31]. In the end, the precursor supported material is dried followed by consecutive heat treatment with inert gas in order to further enhance and stabilise the metal catalyst [32]. Owing to its ease of metal loading control as well as high utilisation of the active component with less metal precursor dosage, the impregnation method is profoundly known as one of the well established procedures for SCR catalyst preparation [32–33]. Jeong et al. [34] applied the impregnation technique to study the characteristics of graphene-supported tungsten (WO_3_) catalyst in a selective catalytic reduction system. The transmission electron microscopy energy-dispersive X-ray spectroscopy (TEM-EDS) measurements in their study showed that the WO_3_ particles were very well distributed over GR and the catalyst surface. The use of a precursor solution (ammonium metatungstate) further enhanced the dispersion of GR, resulting in high catalyst dispersion. Thus, huge amounts of NO could be successfully removed via the catalytic reaction. Qi et al. [35] used the impregnation method to prepare Ce-modified Mn-based catalysts with the aim of studying the effect of a different calcination atmosphere (N_2_, air and O_2_) on the performance of SCR-NH_3_ of NO. Both Ce and Mn particles were well distributed onto the catalyst surface. Nam et al. [36] proposed an impregnation method to investigate the promotional effects of tungsten-modified Mn/Ce/Ti catalysts for a low-temperature SCR reaction. It was found that introduction of W metal into the catalyst helped promote the conversion of NO to gaseous NO_2_, thereby promoting a “fast SCR” reaction to improve low-temperature SCR activity.

The sol–gel method has been used quite frequently for the development of nano-based catalysts. Basically, certain chemical solutions together with a foaming agent (citric acid) is mixed vigorously by stirring under room temperature to produce a homogeneous mixture. Subsequently, the produced sols are dried at a specific temperature for 24 hours in order to obtain xerogel. Then, the xerogel is crushed before being calcined at a desired atmosphere condition and temperature [37]. In the case of catalyst preparation, many researchers have adopted the sol–gel method because: (i) it can form a more even distribution of metal component onto the support material, (ii) it can alter the catalyst porous structure using various foaming agents, (iii) it is able to synthesise the metal-supported catalyst in a single-step process, leading to a unique metal oxide interaction and oxide–oxide interactions that are unreachable otherwise [38]. Xiong et al. [39] prepared magnetic iron–cerium–tungsten mixed oxide pellets using a citric acid sol–gel process assisted by microwave irradiation for the SCR-NH_3_ of NO. They found that the dispersion of both cerium oxide and tungsten oxide onto iron the oxide was improved. In addition, the surface absorbtion capacity of oxygen concentration was enhanced, thus increasing selective catalytic reduction activity. Yao et al. [40] investigated certain physiochemical properties and found that SCR-NH_3_ catalytic performance is influenced by the function of support materials. In their study, SiO_2_ was synthesised using the sol–gel method. Zeng et al. [41] implemented the sol–gel technique to prepare CuO–TiO_2_ catalysts for the selective catalytic oxidation of NO. Based on the results, the CuO–TiO_2_ catalysts demonstrated higher catalytic activity than the Cu_0.07_/Ti catalyst prepared using the impregnation method. The CuO–TiO_2_ catalyst possessed more highly dispersed CuO species as well as many oxygen vacancy active sites, which enhanced the NO gas and made it easily attachable during the catalytic reaction. Similar to previous work, Zhu et al. [42] applied the sol-gel method to study the effect of a particular synthesis method on the catalytic activities of CoO*_x_*–TiO_2_ at low-temperature SCR-NH_3_. In comparison, the CoO*_x_*–TiO_2_ catalyst was also prepared using the impregnation approach. They found that the Co–TiO_2_ catalyst prepared using the sol–gel method had better catalytic activity than that of the impregnation method. Besides that, a huge amount of oxygen vacancies and superoxide ions was observed when cobalt metal was introduced via the sol–gel method, thereby enhancing the SCR performance.

On the other hand, the co-precipitation method has also been applied in the preparation of the SCR catalyst. This technique is much more preferable due to its simple handling procedure and time-saving process. In addition, particle size and composition of the catalyst structure is easy to control [43]. In general, the preparation method starts by initially dissolving a certain amount of metal chemical forms into a homogenous solution. An ammonia solution is then added gradually to the earlier mixture until the pH reaches 10. Then, the slurry is filtered, washed, and dried overnight in the oven before undergoing the activation procedure in the furnace at a specified reaction temperature. Many researchers have used the co-precipitation technique for catalyst preparation to investigate the SCR reaction of NO. For instance, Zhang et al. [44] prepared Mn–FeO*_x_*/CNT catalysts through redox co-precipitation to investigate NO reduction with NH_3_ at low operating temperatures. Excellent SCR activity was observed since the Mn-FeO*_x_*/CNT catalysts possessed high-stability morphology, high chemisorbed oxygen content, as well as strong reducibility at low SCR temperature. Zhang et al. [45] used the co-precipitation method to produce a high loading Zn- (Fe-, Ni-, Cu- or Ag-) promoted Co/Al_2_O_3_ catalyst to study the effect of additive metals on the SCR of NO. From the experimental studies, it was found that this technique definitely contributes towards a good homogeneous dispersion and helps prevent aggregation of highly deposited cobalt on alumina. Moreover, a uniform morphology of the catalyst was obtained and small-sized deposited cobalt was sustained under the presence of Fe, Ni, Cu, Zn or Ag. Meanwhile, Sun et al. [46] planned to enhance the performance of MnO*_x_* catalyst for the SCR-NH_3_ of NO reaction by modifying it with Eu using the co-precipitation method. An enhanced performance of the MnO*_x_* catalyst was observed due to the strong interaction between Mn and Eu, which hinders the MnO*_x_* crystallisation process.

Overall, there are various types of synthesis methods (impregnation, sol–gel, co-precipitation, incipient wetness, and pyridine thermal) that can be used to prepare the SCR catalyst for NO removal. Some of the previously mentioned preparation methods are among the most commonly applied techniques for the SCR-NH_3_ of NO. However, traditional catalyst preparation methods (such as impregnation and co-precipitation) usually result in poor dispersion of the active component and pore blocking on the catalyst surface, thereby decreasing the removal activity of NO [32,47]. Hence, the sol–gel method is seen as the best method for catalyst preparation since it helps achieve better physiochemical properties of SCR catalysts [48].

### Typical characterisation techniques for catalysts

The importance of the characterisation of catalysts for the SCR-NH_3_ reaction for the removal of nitric oxide can be linked specifically to the inner structure of the catalyst. The characteristic features are highly associated with the deposition of carbon onto the catalyst surface, changes of phase, behavior of the catalyst support and active sites, catalyst morphology, type of carbon present on the catalyst, as well as the catalyst chemistry. A broad mixture of characterisation techniques have been applied to characterise the SCR-NH_3_ catalyst, as summarized in the the following [49].

In particular, the catalyst active phases before and after SCR-NH_3_ reaction are typically characterised by:

- atomic absorption spectroscopy (AAS)
- X-ray fluorescence spectroscopy (XRF)
- electron paramagnetic resonance (EPR)
- X-ray photoelectron spectroscopy (XPS)
- temperature-programmed reduction (TPR)
- X-ray diffraction (XRD)

The dispersion of metal, particle size of the catalyst and original state of the metal on the SCR-NH_3_ supported catalysts can be characterised by the following techniques:

- transmission electron microscopy (TEM)
- scanning electron microscopy (SEM)
- X-ray diffraction (XRD)
- CO chemisorption

The behaviour of the SCR-NH_3_ catalyst deposition is typically characterised by:

- temperature-programmed surface reaction (TPSR)
- mass spectroscopy (MS)
- differential thermal analysis (DTA)
- thermogravimetric analysis (TGA)
- temperature-programmed hydrogenation (TPH)
- temperature-programmed oxidation (TPO)

Table 1 summarises the typical characterisation approach for SCR-NH_3_ catalysts.

**Table 1:** Summary of typical characterisation approach for SCR-NH_3_ catalysts.

Characterisation techniques	Catalyst	Aspects characterised or investigated	Refs.

XRD & TEM	Mn/CNT Mn/ZSM-5	average size of active particles on the catalyst surface	[50–51]
NH_3_-TPD	CuO/CNT CeO_2_/CNT	adsorption state of NH_3_ on the surface of the catalyst	[52–53]
TPD	V_2_O_5_/CNT	effect of different catalysts surface on SO_2_ adsorption	[54]
			
H_2_-TPR	Mn/CNT Mn/ZSM-5 CeO_2_/CNT MnO*_x_*/CNT Fe–Cu–O*_x_*/CNT–TiO_2_ CuCe/ZSM-5 CeMo*_x_*O*_y_*/Al_2_O_3_	reducibility of the catalysts	[50–51,53,55–60]
TGA	Mn–Ce/CNT MnO*_x_*/CNT	thermal stability	[55] [57]
XPS	Mn/CNT Mn/ZSM-5 CeO_2_/CNT	surface chemical states and amount of the active component	[50–51,53]
Raman	Mn/CNT	interaction between confined metal species and support material	[50]
NO-TPD & DRIFT spectra	Mn/CNT	interaction of NO with the surface of the catalysts	[50]
XRD	Fe–Cu–O*_x_*/CNT–TiO_2_ V_2_O_5_–WO_3_/CNT Mn–Ce/CNT MnO*_x_*/TiO_2_	crystalline phase and structure of catalyst	[47,56–57,61]
SEM	Fe–Cu–O*_x_*/CNT–TiO_2_ V_2_O_5_–WO_3_/CNT	characterise the morphologies of the samples	[56,61]
XPS	Fe–Cu–O*_x_*/CNT–TiO_2_ Mn–Ce/CNT Ce–MnO*_x_*/TiO_2_ M*_x_*O*_y_*–V_2_O_5_–WO_3_/TiO_2_ CeMo*_x_*O*_y_*/Al_2_O_3_	peak intensity of active metal	[56–60]
NH_3_-TPD	Fe–Cu–O*_x_*/CNT–TiO_2_ V_2_O_5_–WO_3_/CNT M*_x_*O*_y_*–V_2_O_5_–WO_3_/TiO_2_ Ce–MnO*_x_*/TiO_2_ CuCe/ZSM-5	acidic sites in the catalyst	[56,58–59,61–62]
NOx-TPD	Fe–Cu–O*_x_*/CNT–TiO_2_	record the NO and NO_2_ signals	[56]
TEM	Fe–Cu–O*_x_*/CNT–TiO_2_ V_2_O_5_–WO_3_/CNT Mn–Ce/CNT CuCe/ZSM-5	support diameter and metal species particle size	[56–57,61–62]
BET	Mn/ZSM-5 Mn–Ce/CNT Ce–MnO*_x_*/TiO_2_ MnO*_x_*/TiO_2_ M*_x_*O*_y_*–V_2_O_5_–WO_3_/TiO_2_	textural properties of the catalyst (specific surface area, pore volume, and pore size of catalysts)	[47,51,57–59]
TPR	Ce–MnO*_x_*/TiO_2_	influence of metal content on the oxidation states of catalyst	[58]
XRD	M*_x_*O*_y_*–V_2_O_5_–WO_3_/TiO_2_	crystalline phases of the supports and catalysts	[59]
SEM & TEM	M*_x_*O*_y_*–V_2_O_5_–WO_3_/TiO_2_	morphologies and textural features of the catalyst	[59]

### Carbon-based materials as catalyst supports for NO removal

Carbonious materials have rich physical and chemical properties, which lend to their high porosity and good surface chemistry. In line with this, carbon-based materials are found suitable for use in most catalytic process applications. Although carbonious materials have traditionally been used as supports for catalysts in heterogeneous catalytic processes, they are becoming more familiar as catalysts of their own [63–65]. Carbon nanotubes (CNTs), activated carbon (AC), and graphene (GR) are among the most typical choices for carbon-based supports.

Several features can be seen when applying carbon-based materials as a catalyst support. The dispersion of the active phase over the support increases due to the large surface area and high porosity [64,66]. The pore size distribution can be altered to suit the requirements of a specific type of reaction. The surface chemistry of a carbon-based catalyst also plays a significant role, as it influences catalytic performance especially during the synthesis stage. In terms of interactions, carbon-based materials are known to be hydrophobic in nature. These materials normally show a low affinity against polar solvents, such as water, and in other cases, have a high affinity against non-polar solvents, such as acetone. Although the essence of carbon-based materials are mostly likely to be hydrophobic, which will eventually agitate the uniform distribution of the active component onto the carbon support, the surface chemistry of the carbon material could be simply adjusted, for instance, by undergoing an oxidation process, in order to increase the hydrophilicity of the carbon materials. Aside from the easy modification of the porous structure and surface chemistry, carbon-based materials also portray other distinct advantages [64]: (i) easier reduction of metals on the support; (ii) resistance towards acids and bases resulting in best carbon structure; (iii) firm at high working temperature (>750 °C) due to sturdy carbon composition; (iv) able to be produced in different physical shape (granules, pellets, fibres, and cloth) as carbon-based catalyst is very much porous in structure; (v) the cost of carbon-based supports is usually lower and more economical as compared to the conventional supports (silica and alumina) [67].

In recent years, many industrial applications have used carbon as a support material for catalysts. These catalysts are applied during the reaction process of oxidising organic compounds, desulphurisation, halogenation, and as fuel cells [64,66–67]. Unfortunately, all these industrial processes result in an unhealthy environment. Nowadays, treating the emission of nitric oxide, especially from industrial exhaust gas, has become a great concern [54,68–70].

Various types of metal species have been incorporated onto the surface of carbon materials. In order to integrate carbon materials with metal or metal oxide, a functionalisation process known as pre-treatment is required since the surface of pristine CNTs is hydrophobic and inert in nature. Unfortunately, existing preparation methods suffer from scarce deposition, catalyst particle aggregation, and unwanted large-sized metal particles. After attaching the oxide functional group onto the CNTs, the main properties of CNTs are usually affected. As a result, the performance of the as-prepared hybrid material will be impaired. Therefore, more preparation methods with less impact on the properties of CNTs have to be developed. This process is also important for the purification of pristine CNTs from amorphous carbon, fullerenes, coal, and catalyst particles by means of their production. The wet chemical method is considered to be one of the most efficient methods for purification, activation, and functionalisation of CNTs. In this method, CNTs are immersed in a solution containing an oxidising agent for a few hours. A certain amount of heat is also applied to accelerate the oxidation process. The most commonly used technique for surface oxidation is acid treatment such as nitric acid or preferably nitric/sulphuric acid, as this results in the formation of oxygen-containing groups (OH, C=O and COOH) on the surface of the CNTs. The amount of oxygen-containing groups formed depends on the treatment method used. The chemical modification of the surface of (MWCNTs) multiwall carbon nanotubes is required via the oxygenated functional groups for the attachment of metal nanoparticles. It is believed that the oxidative treatment may create defects to the hexagonal or pentagonal structures of the GR sheet in the nanotubes. The functional groups can be introduced at the bent parts, defect sites, and at the sidewall of the MWCNTs. Acid treatment enables the covalent attachment of oxygenated groups on the surface and the open ends of the CNTs. After undergoing pre-treatment, the treated-CNTs will be dispersed with the choice of metal species. In the next step, several synthesis methods are applied such as impregnation, pyridine thermal, sol–gel, and incipient wetness in order to enhance the dispersion of metal element onto the carbon surface. Therefore, a proper preparation technique for the carbon-based catalyst is important to obtain good catalytic performance.

#### Carbon nanotubes as a catalyst support

Nowadays, CNTs are recognised as an attractive catalyst support for SCR catalysts due to their electronic properties and unique nanostructure [71–73]. CNT-supported metal oxide catalysts are well-known in the adsorption field and present interesting properties for the denitrification of NO*_x_* species [74–75]. Ma et al. [56] investigated the NO activity at low operating temperatures on iron-copper-oxide TiO_2_ and CNT (Fe–Cu–O*_x_*/CNT-TiO_2_) catalyst supports prepared via the sol–gel method. The Fe–Cu–O*_x_*/CNT-TiO_2_ catalyst demonstrates the highest (90%) nitric oxide conversion at a reaction temperature of 175 to 275 °C. It is observed that the active component was highly dispersed onto the TiO_2_ and CNT surface because the copper oxide was not detectable by XRD measurements. CNTs play a significant role in the catalyst structure, as they promote the distribution of metal oxides. The XPS analysis shows that the intensity peak of Cu and Fe reduces when the CNTs are added into the catalyst, thus enhancing the conversion of nitric oxide. Moreover, Fe–Cu–O*_x_*/CNT–TiO_2_ catalysts show high resistance towards H_2_O and SO_2_. With the introduction of 10% H_2_O into the inlet gas at 225 °C, the NO*_x_* conversion rapidly decreased from 99% to 65% over the Cu_3_Fe_1_Ti_85_C_5_ catalyst, and then slowly recovered and kept constant at 85%. On the contrary, the activity of NO*_x_* completely recovered and remained consistent during 6 hours of testing, as soon as the H_2_O flow stopped. It was found that the competitive adsorption between the H_2_O molecule and NH_3_ reactant is the cause of reduction in the conversion of NO*_x_* [76]. Meanwhile, the NO*_x_* conversion immediately reduced from 99% to 84% when SO_2_ was present in the inlet gas at 225 °C. Even after the SO_2_ introduction had stopped, the efficiency was still kept low (70%), implying the phenomenon of catalyst deactivation. At 250 °C reaction temperature, NO*_x_* removal also decreased to 81–84%. However, when the SO_2_ supply was switched off, the NO*_x_* recovered up to 88.34%, suggesting a partial recovery of the catalyst deactivation. Phil et al. [77] and Li et al. [78] justified the possible reasons for catalyst deactivation. According to them, this was mostly due to the deposition of sulphates and bisulphates on the catalyst surface. By applying activated carbon (AC) as the catalyst support, the decomposition temperature of these species was reduced [79]. Correspondingly, in the study, the decomposition temperature of both sulphates and bisulphates were reduced with the introduction of CNTs as the support material for the catalyst. Hence, recovery of SO_2_ deactivation was achieved at 250 °C.

Fang et al. [53] found that CeO_2_ particles are highly dispersed on the surface of CNTs when prepared using the pyridine-thermal (PT) route compared to other methods such as impregnation (IM) and physical mixture (PM). The CeO_2_/CNT-PT has smaller and narrower ceria particle size distribution (2 to 14 nm) than CeO_2_/CNT-IM (6 to 20 nm) based on the TEM and HRTEM analysis. In addition, NH_3_-TPD analysis was carried out in order to determine the surface acid amount and strength in the prepared catalysts. It was found that CeO_2_/CNT-PT had much stronger acid sites since the desorption peaks shifted to a higher temperature range, as compared to CeO_2_/CNT-IM and CeO_2_/CNT-PM catalysts [80]. In this moment, the largest area of the desorption peaks can be observed in CeO_2_/CNT-PT, signifying that the amount of NH_3_ adsorbed on the surface of CeO_2_/CNT-PT is greater than on CeO_2_/CNT-IM and CeO_2_/CNT-PM [80–81]. Meanwhile, the XPS analysis indicates that there was more oxygen surface vacancy in the CeO_2_/CNT-PT catalyst, thus, enhancing the NH_3_-SCR reaction. In comparison, Fang et al. [53] studied the performance of the catalysts via the impregnation and physical mixture methods. More than 90% of NO was removed at a reaction temperature between 250 °C and 370 °C for the CeO_2_/CNT-PT catalyst compared to CeO_2_/CNT-IM and CeO_2_/CNT-PM with only 85% and 80% of NO conversion at a reaction temperature below 380 °C, respectively. This suggests that the CeO_2_/CNT-PT catalyst has better catalytic activity.

Wang et al. [57] investigated the use of mixed Mn–Ce oxides-supported CNTs (Mn-Ce/CNT) for NO removal. An array of Mn–Ce/CNT catalysts with different molar ratios (0.6 to 1.8%) were prepared via the incipient-wetness method. During the preparation of the catalysts, the CNTs were pre-treated with concentrated nitric acid for roughly 16 hours. This procedure is advantegous since it helps with the dispersion of mixed oxides onto the surface of CNTs. More than 90% conversion of NO at a reaction temperature between 120 °C to 180 °C was recorded by all Mn(0.4)–Ce/CNT catalysts. Diffraction peaks corresponding to CNTs were the only pattern detected by XRD for all Mn(0.4)–Ce/CNT catalysts. Beside that, negative peaks were observed in the CeO_2_ and MnO_2_ crystalline phase at high loading (1.8%). This suggests that the Mn–Ce particles were evenly distributed on the CNTs. The TEM images specified that the size of Mn–Ce particles were very small crystals and well dispersed on the surface of the MWCNTs. Further analysis was conducted using HRTEM in order to determine the exact sizes and distribution of metal particles. The mixed metal was well distributed with Mn–Ce particle sizes ranging from 2 to 4 nm on the CNT surface. Su et al. [82] investigated the effect of the position of MnO*_x_* particles inside and outside of CNT cavities on the NO activity in the SCR reaction. It is found that the MnO*_x_* particles inside the CNTs displayed higher NO activity in the SCR reaction compared to when the MnO*_x_* particles were introduced outside of the CNT surface. In line with this research, Wang et al. [57] found that the high catalytic performance by Mn–Ce/CNT catalyst was partly attributed to the Mn–Ce particles found inside the cavity of the CNTs.

Wang et al. [83] reported high NO conversion (≈90%) at a relatively high reaction temperature of 200 to 300 °C on the Mn–Ce/CNT catalyst prepared using the incipient-wetness method. To improve the nitric oxide activity, especially at lower temperature, an alternative approach known as the liquid-phase method was applied in preparing the Mn–CeO*_x_*/CNT catalyst [83]. Interestingly, excellent NO activity at low reaction temperatures was demonstrated by the developed catalysts. Even at a low catalytic temperature of 80 °C, the 63–85% NO conversion was still achieved. Soon after, the NO conversion started to rise significantly, nearly up to 100%, as the reaction temperature increased from 120 to 180 °C. The authors also investigated the Mn-CeO*_x_*/CNT catalysts at different Mn/(Mn + Ce) molar ratios (0.4 to 0.6) on the NO removal activity. 0.5 Mn/(Mn + Ce) molar ratio was found to be the optimum loading amount for Mn–CeO*_x_*/CNT catalyst preparation. From the HRTEM images, an uneven shape and fuzzy crystal lattice was identified on the metal nanoflakes suggesting that the Mn–CeO*_x_*/CNT catalyst is amorphous in structure. The XRD result confirms only the presence of typical CNT peaks. Both of these features highly contribute towards high NO activity at low SCR temperature. This conclusion is in agreement with the research work of Tang et al. [84], where amorphous oxides showed greater NO activity compared to oxides with a crystalline structure in SCR at low temperature.

The effect of thermal treatment condition over MnO*_x_*/CNT catalysts for the SCR of NO was investigated by Wang et al. [55]. The conventional method for CNT-based catalyst preparation always takes place in an inert atmosphere at a high calcination temperature. However, this condition is undesirable since it could disturb the NO activity due to the catalyst mass loss at high calcination temperature [57]. Hence, Wang et al. [55] prepared the MnO*_x_*/CNT catalysts based on three different thermal conditions; MnO*_x_*/CNT-A1 (calcined in air, 250 °C for 2 h), MnO*_x_*/CNT-A2 (calcined in air, 300 °C for 50 min), and MnO*_x_*/CNT-N3 (calcined in N_2_, 300 °C for 2 h) to examine their catalytic activity in the NO removal reaction. It was found that MnO*_x_*/CNT-A1 (calcined in air at 250 °C for 2 h) demonstrated the highest catalytic activity compared to the sample calcined in air at 300 °C for 50 min and the MnO*_x_*/CNT catalysts (calcined in N_2_ at 300 °C for 2 h) throughout the entire temperature window. Even at a low reaction temperature of 80 °C, the recorded NO conversion was up to 60%. However, the NO activity declined in both the MnO*_x_*/CNT-A2 (calcined in air at 300 °C for 50 min) and MnO*_x_*/CNT-N3 (calcined in N_2_ at 300 °C for 2 h) catalysts. This is greatly associated with the CNTs reducing MnO*_x_* to a lower valence state during the calcination process. Thus, thermal treatment condition plays a vital role in obtaining the best NO removal for CNT-based catalysts in the SCR reaction.

Su et al. [50] modified the catalytic properties of (Mn) species by introducing MnO*_x_* either on the outer CNT surface or on the inner and outer CNT surface for the SCR of NO with NH_3_. The recorded NO*_x_* conversion was very low over blank CNTs at a reaction temperature ranging from 80 °C to 300 °C. By introducing a small amount of Mn (3%) to the CNTs, the catalytic activity of MnO*_x_*/CNTs was improved tremendously at a reaction temperature between 150 and 250 °C. This positive performance may be greatly associated with the oxidation state of Mn species as well as the interaction type present between the MnO*_x_* species and the surface of CNTs due to the unique structure of the CNTs [85]. According to the H_2_-TPR result, MnO*_x_*/CNTs with Mn content of 3% showed a two-step reduction process, as some of the MnO*_x_* was introduced inside the CNT cavities. Meanwhile, the reduction temperature was identified to be at 219 °C and 418 °C. Meanwhile, the 3% Mn-out-CNTs_400_ peaks were around 440 to 520 °C. Generally, the valance state and electron environment of manganese are two key components that affect the reduction steps and reduction temperature [50]. Previous work done by Cao et al. [86] showed that the amount of electrons found on the inner part of the CNT surface was insufficient compared to the one located on the outside surface. When the metal oxide species are introduced at the side, which lacks electrons, metal oxide will tend to donate more electrons, leading towards easier reduction activity. Other reports by Chen et al. [87–88] reveal that the reduction of Fe_2_O_3_ nanoparticles, which are located inside the cavity, occur at 600 °C while the reduction of the outer surface of the MWCNTs occur at 800 °C. Therefore, the difference in reduction activity may be associated with the strong interaction between MnO*_x_* nanoparticles with the inner and outer CNT surface. Concurrently, the number of oxygen molecules could be supplied when MnO*_x_* is introduced onto the CNT surface, promoting high SCR activity.

In another work, Zhang et al. [70] compared the catalytic activity of Mn–Ce species on CNT using an in situ (reflux) method. In this method, poly(sodium 4-styrenesulonate) (PSS) was used as surfactant during the synthesis of the catalyst in order to assist with the dispersion of CNTs in aqueous solution and to maintain its stability. From the experimental activities, it was found that the MnCe@CNTs-R catalyst showed the highest (>90%) NO conversion and most comprehensive reaction temperature (200–350 °C) in comparison with other catalysts prepared using impregnation and mechanically mixed methods. Both MnCe@CNTs-I and MnCe@CNT-M prepared by impregnation and mechanically mixed methods showed a NO conversion of only up to 90% and 85%, respectively, at a 170 °C and 200 °C temperature window. Upon undergoing the characterisation study, the XRD and TEM results showed that prepared MnCe@CNTs-R afforded an excellent dispersion amount of active species on the CNT surface. This phenomenon could be one of the main reasons favouring the outstanding NO activity. Meanwhile, the XPS analysis revealed that the prepared MnCe@CNTs-R catalyst had more active sites, which promoted the NH_3_-SCR reaction since more Mn atoms were exposed onto the CNT surface. Besides that, the oxidation process helped oxidised NO to NO_2_. This was due to the interaction present between the MnO*_x_* and CeO*_x_* species, resulting in more oxygen vacancy in the MnCe@CNTs-R catalyst. This rapidly enhanced the SCR reaction, making it faster. The NH_3_-TPD analysis shows that the MnCe@CNTs-R catalyst was not only able to demonstrate a large amount of acid content, but also a stronger acid strength. In terms of stability, the MnCe@CNTs-R activity remained constant during the test period (300 °C). However, the NO conversion for both MnCe@CNTs-I and MnCe@CNTs-M slowly reduced over time. Furthermore, MnCe@CNTs-R exhibited high tolerance towards SO_2_ and H_2_O.

Bai et al. [52] studied CuO supported on CNTs. The catalyst was developed to better understand the catalytic behaviour of CNT-based catalysts especially in reducing the amount of nitric oxide in an SCR reaction. Different amounts of CuO loadings were studied at a 200 °C reaction temperature. Apparently, the catalytic activity of the CuO/CNT catalyst was observed to rise as the CuO loading increased from 1 to 10 wt %. About 88.5% of NO was converted when 10 wt % of CuO loading was introduced into the CNT. Unfortunately, the NO conversion decreased as the amount of CuO loading became higher than 10 wt %. It is surmised that the reduction in NO activity is mostly due to the aggregation of CuO particles on top of the CNT surface [54]. The catalytic performance of 10 wt % CuO/CNT was also studied over a 120 to 150 °C temperature window. The NO conversion over 10 wt % CuO/CNT catalyst increased as the reaction temperature increased. Approximately 88.5% of NO was converted when the reaction temperature increased to up to 200 °C. However, the conversion activity of NO reduced to 70.5% when the reaction temperature increased to 250 °C. This result is in accordance with the tendency of NH_3_ to oxidise at high reaction temperature, leading to a lower reduction of NO activity [54]. In terms of stability, 10 wt % CuO/CNT catalyst was highly stable at 200 °C temperature where a great NO conversion efficiency of 88.5% as well as outstanding catalytic activity at 72 h of running was also observed. All the features above are just some of the main advantages of using the CuO/CNT catalyst in the SCR of NO at low temperature.

Bai et al. [54] investigated the activity of V_2_O_5_/CNT catalysts at a low SCR reaction temperature. They found that V_2_O_5_/CNT catalysts have higher catalytic activity compared to V_2_O_5_ particles supported on AC (V_2_O_5_/AC) at 200 °C reaction temperature. The BET analysis confirms that the CNTs have a lower (220 m^2^/g) surface area than AC (615 m^3^/g), indicating that the catalytic properties in CNTs are better than AC because of the former’s special structural features [54]. Meanwhile, a study on the effect of SO_2_ shows that there was no significant conversion of nitric oxide by V_2_O_5_/AC either with or without the presence of SO_2_ in the reaction stream. However, the conversion of nitric oxide momentously increased and became almost linear at a high level of SCR reaction by V_2_O_5_/CNT (0.1–1 wt %) catalysts in the presence of SO_2_. This trend implies that the presence of SO_2_ has helped increase the reduction of NO over V_2_O_5_/CNT catalysts, especially at low SCR temperature. Bai et al. [54] added that the V_2_O_5_ loading is the sole factor influencing the promotional effect of SO_2_ in the system. Hence, there was a synergistic connection among CNTs and the vanadium species in the catalyst. Unlike in any other study, SO_2_ usually displays a poisoning rather than a promoting effect. In this study, the SO_2_ promoting effect is more likely associated with the formation of SO_4_^2−^ species on the catalyst surface. These SO_4_^2−^ species would act as new acidic sites that help improve NH_3_ adsorption, thereby promoting the activity of the catalyst. Similar observations were also reported elsewhere [89–91]. Therefore, the V_2_O_5_/CNT catalyst is justified as one of the most promising catalytic materials for SCR at low temperature due to the unique promoting effect of SO_2_.

Wu et al. [61] introduced the idea of implementing CNTs, a by-product obtained from the prior hydrogen production, as supports for the V_2_O_5_–WO_3_ catalyst. In their study, the catalysts were prepared via the impregnation method, while ammonia was used as a reducing agent. A characterisation study conducted using TEM analysis revealed that the diameter of the CNTs was around 50 nm, whereas the V_2_O_5_–WO_3_ particle size was about 8 nm. A similar observation was found by Su et al. [50]. In their study, Mn/CNT catalysts were used in the SCR activity. The size of the Mn particles was found to be around 8 nm, while the CNT diameters were around 50 nm. Huang et al. [92] used V_2_O_5_ supported on CNTs as the SCR catalyst. Upon study, the size of the metal particle was found to be around 10 nm, while the diameter of the CNTs was between 10 to 100 nm. Meanwhile, the XRD analysis showed that tungsten oxides could not be identified because their particle size was too small to be determined [93]. This indicates that the WO_3_ particles were evenly dispersed on top of the catalyst surface [94]. In terms of NO*_x_* reduction efficiency, the V_2_O_5_–WO_3_/CNT catalyst achieved more than 95% conversion in reaction temperatures of 340 to 400 °C. However, low NO*_x_* activity was observed when the reaction temperature went below 200 °C, which is probably due to the lack of Lewis active sites. Hence, further investigations into lowering the SCR reaction temperature with high NO*_x_* productivity were proposed. A summary of the reaction conditions and NO activity for CNT-based catalyst supports is shown in Table 2.

**Table 2:** Summary of the reaction conditions and NO activity for CNT-supported catalysts.

Catalyst	Preparation method									
	Activation condition									
	Reaction condition							Best NO removal		Refs.
	NO (ppm)	NH_3_ (ppm)	O_2_ (%)	SO_2_ (%)	Balance (gas)	Flow rate (mL/min)	GSHV (h^−1^)	Conversion (%)	Temperature (°C)	

Fe–Cu–O*_x_*/CNT–TiO_2_	sol–gel									
	calcination: 500 °C/4 h (N_2_)									
	550	550	5	–	N_2_	500	36,000	99	175–250	[56]
V_2_O_5_–WO_3_/CNT	impregnation									
	calcination: 450 °C/3 h (N_2_)									
	0.06 (vol %)	0.06 (vol %)	3	–	N_2_	300	–	>95	340–420	[61]
CeO_2_/CNT-PT CeO_2_/CNT-IM CeO_2_/CNT-PM	PT-pyridine thermal route (main), IM-impregnation, PM-physical mixture									
	calcination: 500 °C/4 h (N_2_)									
	500	500	3	100	N_2_	250	20,000	>90	250–370	[53]
Mn-Ce/CNT	incipient wetness									
	calcination: 300 °C/1 h (air)									
	500	500	5	–	N_2_	700	84,000	>90	120–180	[57]
MnCe@CNT-R MnCe@CNT-I MnCe@CNT-M	R-reflux (main), I-impregnation, M-mechanical									
	calcination: 500 °C/6 h (N_2_)									
	500	500	3	100	N_2_	–	10,000	>90	200–350	[70]
Mn–CeO*_x_*/CNT	liquid-phase									
	vacuum drying: 120 °C/12 h									
	500	500	5	–	N_2_	700	30,000	≈100	120–180	[83]
MnO*_x_*/CNT-A1 MnO*_x_*/CNT-A2 MnO*_x_*/CNT-N3	incipient wetness									
	A1 – calcination: 250 °C/2 h (air), A2 – calcination: 300 °C/50 min (air), N3 – calcination: 300 °C/2 h (N_2_)									
	500	500	5	–	N_2_	700	38,000	>60	80–180	[55]
Mn-in-CNTs Mn-out-CNTs Mn-both-CNTs	wet chemical									
	in – ethanol; ultrasonic treatment/4 h; calcination: 300–400 °C (N_2_), out – xylene; ultrasonic treatment/3 h; calcination: 300–400 °C (N_2_), both – xylene; ultrasonic treatment/1 h; calcination: 300–400 °C (N_2_)									
	0.08 (%)	0.08 (%)	5	–	Ar	600	35,000	>90	200–250	[50]
CuO/CNT	pore volume impregnation									
	calcination: 250 °C/2 h (Ar)									
	450	500	5	–	N_2_	–	30,000	88.5	200	[52]
V_2_O_5_/CNT	pore volume impregnation									
	calcination: 500 °C/5 h (argon steam)									
	450 (µ/L)	500 (µ/L)	5	–	N_2_	–	30,000	≈100	250	[54]

#### Activated carbon and activated carbon fibre as a catalyst support

The discovery of CNTs, AC, and activated carbon fibre (ACF) as catalysts and catalyst supports has been the focus of researchers in NO removal application [95–96]. The adsorption ability of both AC and ACF depends highly on its surface area, internal pore structure, surface characteristics, and the presence of functional groups on the pore surface [97]. Tsen et al. [95] evaluated the catalyst performance of coconut shell AC as a support with several active metals such as Cu, Ni, Co, and Fe. The results conclude that NO conversion increased with an increasing atomic number of active components. Sumathi et al. [2] prepared palm shell AC impregnated with several metal oxides (Ce, Ni, Fe, and V). The palm shell support was found to be capable of removing NO gas and further loading with metal oxides increased the conversion reaction due to the increased micropore volume of the palm shell AC. Yoon et al. [98] studied the effect of propellant waste on ACF by impregnation and heat treatment methods. The introduction of a nitrogen functional group by propellant waste promoted the activity removal of NO two-fold to that of the unmodified ACF at low temperature. The SEM analysis reveals that propellant waste was fully oxidised on the ACF surface after being treated at 500 °C. Meanwhile, Zhu et al. [96] modified ACF through low-temperature oxygen plasma and nitric acid, which are respectively denoted as activated carbon modified by plasma (ACFP) and activated carbon modified by nitric acid (ACFN). The results indicate that the CeO_2_ species on ACFN produced higher activity than those on ACFP. This is because the surface of CeO_2_/ACFN is more acidic as it was treated with nitric acid and the reaction between NO and the catalyst surface became more active. Using several characterisation techniques, such as Fourier transform infrared spectroscopy (FTIR), X-ray photoelectron spectroscopy (XPS), and temperature-programmed desorption (TPD), the comparison of the properties for both ACFP and ACFN was clarified. Chuang et al. [18] considered three different methods, i.e., impregnation, polyol, and microwave-heated polyol methods for the synthesis of an economical Cu/AC catalyst. Among these methods, the microwave-heated polyol method showed the highest catalytic activity for the Cu/AC catalyst with 80.1% conversion of NO. Analysis using FESEM verified that the Cu particles on the AC surface were better dispersed compared to the catalysts prepared using the other two methods. Interestingly, the MnO_2_/ACF catalyst prepared by the co-precipitation method appeared to be a promising catalyst, as it can reduce NO at a temperature as low as 25 °C [1]. A similar finding was reported by Sousa et al. [99], where the melamine/AC catalyst prepared by impregnation removed NO at a reaction temperature of 25 °C with a high conversion of 88%. This suggests that the NO removal by AC and ACF could be a serious contributor to sustainable economic growth by offering cost-effective low energy consumption. Although AC and ACF are extremely effective at removing NO due to the combination with certain active components, the investigation into carbon-based supports is still in progress due to some limitations such as the presence of O_2_, reactivity at low temperature, and also the components of the carbon itself.

Other unique catalyst modifications have also be implemented, especially improvements in the quality of supporting materials. Klinik et al. [100] treated activated carbons with N-compounds and further enhanced the catalyst surface chemical properties with manganase oxides. It was observed that the addition of water into the SCR system reduced NO activity by 10 to 20% but increased the selectivity of N_2_. The N-AC/MnO*_x_* catalyst can be justified as one of the promising catalysts for SCR-NH_3_ reaction since it yields the highest amount of NO conversion (≈100%) at an operating temperature between 180 to 220 °C. Boyano et al. [101] prepared a series of vanadium-loaded carbon-coated monoliths treated with different concentrations of acids. The aim was to investigate the effect of distinct oxidative treatment conditions on the NH_3_-SCR catalytic environment including chemical surface and texture properties. HNO_3_ treatment resulted in the most outstanding performance. The vanadium-loaded carbon treated with HNO_3_ acid achieved the highest NO reduction activity, as there was a large amount of carboxyls and lactones present on the catalyst surface. Acid treatment is an important factor that could enhance the performance of NO activity since it is closely related to the density of the surface oxygen group obtained during the oxidation process. Muniz et al. [102] implemented Nomex-based carbon fibres and Kevlar-based carbon fibres for the SCR of NO with NH_3_ at low reaction temperature. It was found that the Nomex-based carbon fibres demonstrated a higher degree of NO reduction compared to Kevlar-based carbon fibres due to the presence of the oxygen surface group, which enhances the conversion activity of NO. Lazaro et al. [103] applied vanadium supported onto various forms of AC (powder, briquettes, and monoliths) catalysts via the impregnation method for the SCR of NO with NH_3_. All these AC-based catalysts are porous in structure, which plays an important role for achieving high NO removal activity. Galvez et al. [104] prepared a series of activated carbons doped with vanadium compounds, which successfully achieved considerable reduction activity of NO in the presence of NH_3_ at a reaction temperature between 100–300 °C. A higher NO reduction activity was observed when using a pre-oxidised (SCA750) carbon support. Huang et al. [105] prepared various samples of carbon catalysts from phenol-formaldehyde resins. Each catalyst sample was impregnated with different amounts of *m*-phenyl-enediamine (MPDA) in order to enhance the performance of NO reduction. Upon impregnation of the carbon catalysts with nitrogen, it was observed that the NO catalytic activity increased significantly at operating temperatures below 140 °C. Table 3 summarises the reaction conditions and NO activity for activated carbon and activated carbon fibre-based catalyst supports.

**Table 3:** Summary on the reaction conditions and NO activity for activated carbon and activated carbon fiber supported based catalysts.

Catalyst	Preparation method								
	Activation conditions								
	Reaction conditions						Best NO removal		Refs.
	NO (ppm)	NH_3_ (ppm)	O_2_ (%)	Balance (gas)	Flow rate (mL/min)	GSHV (h^−1^)	Conversion (%)	Temperature (°C)	

CuO*_x_*/AC	wet impregnation								
	calcination: 400 °C/2 h (N_2_)								
	700	700	6	N_2_	500	7,500	62	250	[78]
Fe/AC	volume impregnation								
	calcination: 450 °C/4 h (N_2_)								
	600	–	6	N_2_	500	40,000	95	250	[95]
CeO_2_/ACFP^a^	impregnation								
	calcination: 350 °C/6 h (N_2_)								
	1000	1000	5	N_2_	–	11,000	86	180	[96]
CeO_2_/ACFN^b^	impregnation								
	calcination: 350 °C/6 h (N_2_)								
	1000	1000	5	N_2_	–	11,000	94	180	[96]
V_2_O_5_/AC	impregnation								
	calcination: 500 °C/5 h (Ar)								
	450	500	5	N_2_	1200	–	94	150	[106]
Cu/AC	impregnation, polyol, microwave heated								
	calcination: 300 °C/4 h								
	400	400	6	N_2_	500	43,750	52.7 79.3 80.1	200 200 200	[18]
Cu/AC	co-precipitation								
	calcination: 800 °C/30 min (N_2_)								
	1500	–	5	N_2_	–	6,000	>80	300	[107]
MnO_2_/ACF^c^	co-precipitation								
	calcination: 200 °C/2 h (N_2_)								
	50	–	21	N_2_	–	–	30.6	25	[1]
N-AC^e^/MnO*_x_*	impregnation								
	calcination: 220 °C/50 min (He)								
	800	800	3	He	100	–	≈100	180–220	[100]
CeO_2_/ACF^c^ La_2_O_3_/ACF^c^	Impregnation								
	Calcination: 360 °C/2 h								
	500	–	21	N_2_	266.7	–	70 90	150 350	[108]
Ce/ACF^c^	impregnation								
	calcination: 500 °C/4 h (Ar)								
	150	150	5.6	N_2_	250	–	80	300	[109]
Pd/AC	impregnation								
	reduction: 50 °C/30 min (He)								
	500	–	–	N_2_	–	15,000	>90	100	[110]
melamin/AC	impregnation								
	calcination: 600 °C/50 min (N_2_)								
	1000	–	20	N_2_	–	–	88	25	[99]
propellant/ACF^c^	impregnation								
	calcination: 500 °C/1 h (N_2_)								
	900	–	10	N_2_	500	–	>90	30	[98]
NH_3_/AC	impregnation								
	calcination: 750 °C/1 h (N_2_)								
	300	–	20	N_2_	–	–	70	450	[111]
CeO_2_−Cu−CNF^d^ACF^c^	impregnation								
	calcination: 200 °C/4 h (N_2_)								
	500	–	20	N_2_	–	–	80	30	[112]
Nomex-based carbon fibres Kevlar-based carbon fibres	carbonization								
	carbonized: 700 °C/1 h (N_2_)								
	700	800	3	Ar	300	–	40–80	300–400	[102]
V/AC-powder V/AC-briquettes V/AC-monolith	impregnation								
	activation: 750 °C/2 h (H_2_O or CO_2_)								
	800	800	3	He	100	–	28–88	150	[103]
carbon-SCA750/V_2_O_5_	stirring								
	stirring: ambient temperature/4 h								
	1000	1500	3.5	Ar	–	–	>80	200	[104]
PFC^f^	impregnation								
	heating in vacuum: 60 °C/2 h to 120 °C/12 h								
	500	640	1	He	–	–	11	<140	[105]

^a^Activated carbon modified by plasma; ^b^activated carbon modified by nitric acid; ^c^activated carbon fiber; ^d^carbon nanofiber; ^e^nitrogen promoted activated carbon; ^f^carbon catalysts from phenol-formaldehyde resins.

#### Graphene as a catalyst support

Graphene (GR) is a new outstanding carbon nanomaterial, which demonstrates excellent physiochemical properties such as good thermal conductivity, chemical stability, large surface area, high electron exchange, and a flexible structure [6]. Therefore, GR is considered to have good conductivity at room temperature due to the freely moving electrons on the surface, which can improve the redox performance of the catalyst as well as the catalytic activity in de-NO*_x_* [113]. You et al. [6] verified that high NO conversion can be achieved over GR at low reaction temperature. A MnO*_x_*–CeO_2_/GR catalyst prepared via the hydrothermal method yielded high catalytic activity of almost 100% at 120 °C. The Raman spectra reveal that GR offers more active sites and improves the interaction between MnO*_x_* and CeO_2_ particles on the flexible GR surface. In fact, Guo et al. [114] explains that the removal of NO can be conducted at room temperature with the addition of graphene oxide (GO) onto carbon nanofibres (CNFs). Xiao et al. [115] used GR to obtain high NO conversion at low reaction temperature. Their results indicate that the MnO*_x_*–CeO_2_/GR catalyst achieved higher NO conversion compared to pure MnO*_x_*/GR and CeO_2_/GR. The XPS analysis revealed that the addition of GR could change the composition of the Mn species, which plays an important role in electron mobility and thus leading to low reaction temperature in SCR. Moreover, MnO*_x_*–CeO_2_/GR (0.3%) at 180 °C also showed high catalytic activity when 10 vol % H_2_O and 200 ppm SO_2_ were added to the system. The same catalytic properties, which convey good resistance towards H_2_O and SO_2_ poisoning, was also reported by Lu et al. [116]. The researchers employed Ce–Mn/TiO_2_–GE catalysts prepared via the sol–gel and ultrasonic impregnation methods. It was noted that the hydrophobic groups present on the GR surface are not favourable for H_2_O adsorption. Although some researchers achieved good results for catalyst stability and high H_2_O and SO_2_ resistance, further investigation is still needed to ensure the long-term activity of the prepared catalysts. In addition, there are still limited reports that focus on GR applications for low-temperature SCR. Therefore, there is still a crucial need for more research on catalysts that can provide a reaction temperature as low as room temperature with high NO conversion. Table 4 shows a summary of the reaction conditions and NO activity for graphene-based catalyst supports.

**Table 4:** Summary on the reaction conditions and NO activity for graphene supported based catalysts.

Catalyst	Preparation method								
	Activation conditions								
	Reaction conditions						Best NO removal		Refs.
	NO (ppm)	NH_3_ (ppm)	O_2_ (%)	Balance (gas)	Flow rate (mL/min)	GSHV (h^−1^)	Conversion (%)	Temperature (°C)	

CuO*_x_*/graphite	wet impregnation								
	calcination: 400 °C/2 h (N_2_)								
	700	700	6	N_2_	500	7,500	45	250	[78]
GE/CNF (PGCNF)^a^	electro-spinning								
	activation: 850 °C/10 min (NH_3_)								
	50	–	21	N_2_	–	–	32.83	30	[114]
ACNF^b^ GPNF-1900^c^ GPNF-2400^c^	electro-spinning								
	activation: 800 °C/30 min (N_2_) graphitized: 1900 °C/10 min (N_2_) graphitized: 2400 °C/10 min (N_2_)								
	20	–	21	N_2_	–	–	11 38 45	30 30 30	[117]
MnO*_x_*/TiO_2_−GE^d^	ultrasonic impregnation								
	calcination: 450 °C/6 h (N_2_)								
	500	500	7	Ar	–	67,000	>93	180	[113]
MnO*_x_*-CeO_2_/GR^d^	hydrothermal								
	calcination: 400 °C/2 h (Air)								
	500	500	5	N_2_	–	24,000	≈100	120	[6]
CeO*_x_*–MnO*_x_*/TiO_2_–GE^d^	ultrasonic Impregnation								
	calcination: 450 °C/6 h (N_2_)								
	500	500	7	Ar	–	67,000	>99	180	[116]
MnO*_x_*/TiO_2_–GO^e^	ultrasonic Impregnation								
	calcination: 450 °C/3 h (N_2_)								
	500	500	7	Ar	600	67,000	>95	180	[118]
MnO*_x_*–CeO_2_/GR^d^ MnO*_x_*/GR^d^ CeO_2_/GR^d^	hydrothermal (130 °C/12 h)								
	calcination: 400 °C/2 h (N_2_)								
	500	500	5	N_2_	–	24,000	100 80 20	140 140 140	[115]
MnO*_x_*/GR^d^	hydrothermal (160 °C/24 h)								
	calcination: 400 °C/2 h (N_2_)								
	0.06%	0.06%	3	Ar	500	45,000	>90	190	[119]

^a^Polyacrylonitrile graphene oxide nanofibers; ^b^activated carbon nanofibers; ^c^graphite carbon nanofibers; ^d^graphene; ^e^graphene oxide.

### Non-carbon-based materials as catalyst supports for NO removal

Apart from carbon-based supports, non-carbonious-based materials have also been applied as catalyst supports for nitric oxide removal, especially in the SCR process. Carbon supported materials tend to vaporise easily. This phenomenon would retard the material capability especially during oxidation and hydrogenation reactions [67]. The reproducibility of AC-based catalysts is low since different batches of materials would result in different amounts of ash content [120]. Today, researchers tend to focus on different compound oxides for catalyst support since they possess great features that would result in the higher removal of nitric oxide. Zeolite Socony Mobil–5 (ZSM-5) [62], TiO_2_ [37,47,58–59], and Al_2_O_3_ [60] are some of the examples of the most frequently used catalyst supports. The ZSM-5 support is reported to have a remarkable ion-exchange capacity [121]. Furthermore, the TiO_2_ support is known for its high resistance to sulphur poisoning and sulphates, which are much more stable on the TiO_2_ surface compared to other oxides [47]. In the next sections, a comprehensive review on the non-carbon-based catalyst focusing on the types of supports for SCR reaction is discussed in detail.

#### ZSM-5 as a catalyst support

Nowadays, researchers prefer to modify a currently popular ZSM-5 catalyst support by introducing a second metal in order to broaden the temperature window for the SCR reaction. Usually, a second metal is used as an additive. It was found that the interaction between both metals would significantly influence the catalyst surface physiochemical structure [122–123]. Ceria is one of the most popular promoters used in metal-containing ZSM-5 catalysts. Many studies have implemented ceria due to its exceptional properties, such as: (i) strong metal-support interaction that helps increase the dispersion and stability of the metal catalyst; (ii) formation of oxide ion storage and labile oxygen vacancies; (iii) enabling the easy exchange of oxygen molecule with the medium, as the redox couple between the trivalent and tetravalent oxidation states of the ceria ions are present [124–125]. Pang et al. [126] reported the high activity and stability of the Ce–Cu/ZSM-5 catalyst. However, no attention has been given towards the investigation of redox properties, surface state, or the synergistic effects between Cu and Ce supported on ZSM-5 in the SCR reaction. Recently, Dou et al. [63] modified Cu/ZSM-5 by introducing Ce into the catalyst. Their main target was to actually develop a catalyst that can broaden the temperature window by focusing on the influence of cerium introduction onto the catalyst structure, acid sites, redox property, and chemical state of Cu and Ce species before and after the SCR reaction takes place. The influence of cerium content on the Cu/ZSM-5 catalyst was evaluated by setting the copper content to 2.0 wt %, and varying the cerium content from 0, 0.5, 1.0, 1.5 to 2.0 wt %, corresponding to Cu-Z, CuCe1-Z, CuCe2-Z, CuCe3-Z, and CuCe4-Z, respectively. The experimental results confirm that the temperature window widened to low and high level temperatures when Ce was introduced into the Cu/ZSM-5 sample. Of all the CuCe/ZSM-5 catalysts, CuCe4-Z (where 4 refers to 2.0 wt % of cerium) demonstrated the best catalytic activity with 95% NO being reduced at a 149–427 °C reaction temperature. In terms of catalytic behaviour, the addition of cerium also helped increase the dispersion of copper onto the support surface (ZSM-5) and also prevented crystallisation, which is prone to happen in SCR reactions. In addition, the redox properties of CuCe/ZSM-5 catalysts were also improved with the addition of cerium as the second metal. CuCe/ZSM-5 also showed a higher amount of Cu valence and lattice oxygen compared to the Cu/ZSM-5 catalyst, which are shown from the XPS and H_2_-TPR analysis.

Lou et al. [51] developed a series of Mn/ZSM-5 catalysts for the SCR of NO with ammonia via the precipitation method. The catalysts were prepared at various calcination temperatures (200 to 700 °C), denoted as MnZ-2 (200 °C), MnZ-3 (300 °C), MnZ-4 (400 °C), MnZ-5 (500 °C), MnZ-6 (600 °C), and MnZ-7 (70 °C), respectively. The calcination temperature was found to have a significant effect on the NO catalytic activity. If the calcination temperature is too low, the metal salts will tend to decompose incompletely, resulting in a decrease in the amount of active component on the catalyst surface. On the other hand, if the calcination temperature is too high, sintering could occur in the catalyst, which later leads to a serious decrease in the catalyst surface area as well as reduction in the even distribution of active component onto the catalyst [51]. Generally, the calcination temperature is considered to be associated with the manganese oxide final oxidation states, crystallinity, and surface area, which can help determine the success of the NO reduction activity [51]. Limited studies have been done to explore the influences of calcination temperature on Mn/ZSM-5 catalysts. Still, detailed studies on how calcination temperature affects the formation of different MnO*_x_* species in SCR activities have not yet been reported. Therefore, Lou et al. [51] studied the variation of calcination temperature of Mn/ZSM-5 catalysts in order to analyse its effects on the physical and chemical properties of Mn/ZSM-5 catalysts. Mn/ZSM-5 catalysts that were prepared at lower calcination temperatures (<500 °C) resulted in higher Mn surface and lattice oxygen concentration, as well as a larger surface area. Moreover, lower calcination temperatures (<500 °C) promote the SCR Mn/ZSM-5 catalyst activity since the formation of Mn_3_O_4_ species and amorphous MnO_2_ species will react together in the NO reduction process. On the contrary, a higher calcination temperature (600 to 700 °C) reduced the number of Mn concentration, resulting in smaller surface area and increased crystallinity of the catalysts. This phenomenon will lead to the formation of crystalline Mn_2_O_3_ species, thus reducing the SCR process. Overall, the MnZ-3 (calcined at 300 °C) catalyst demonstrated the best performance of NO reduction out of all the Mn/ZSM-5 catalysts. Almost 100% of NO was converted at a reaction temperature of 150 to 390 °C.

#### TiO_2_ as a catalyst support

Recently, investigations into bimetallic catalysts such as Fe–Mn/TiO_2_ [47,58], Mn–Ni/TiO_2_ [127], Ce–W/TiO_2_ [128], and ternary mixed-oxide catalysts such as Nb–Ce–W/TiO_2_ [129] and Zr–V–W/TiO_2_ [130] supported on TiO_2_ have increased in popularity among scientists. These research studies imply that all these catalysts demonstrate high catalytic activities. For example, the Mn–Ni/TiO_2_ [127] and Fe–Mn/TiO_2_ [47,58] catalysts showed almost 100% conversion of NO at low temperature (200 °C) reaction conditions. In addition, the remarkable thermal stability of bimetallic and ternary catalysts has also been recognised. Shi et al. [130] found that the aged Zr–V–W/TiO_2_ catalyst is more active than the fresh catalyst at a reaction temperature of 150 to 400 °C, after undergoing thermal aging at 750 °C for 12 h. Lee et al. [131] outlined the applications and synthesis of advanced metal oxide catalysts. They reported that the SCR activities and selectivity were greatly improved with the addition of cerium. This is due to the good thermal stabilities as well as high activities portrayed by the catalysts. Zhang et al. [59] studied the ternary mixed-oxide catalysts for the SCR-NH_3_ of NO reaction. A partial substitution method of metal oxide (M*_x_*O*_y_*, M = Fe, Co, Ni, Cu, Sr, La, and Ce) for V_2_O_5_ in the V_2_O_5_–WO_3_/TiO_2_ catalyst was prepared in accordance to the preparation of ternary mixed-oxide catalysts. The developed M*_x_*O*_y_*–V_2_O_5_–WO_3_/TiO_2_ catalysts were then tested for SCR-NH_3_ reaction. At a reaction temperature of above 400 °C, the M*_x_*O*_y_*–V_2_O_5_–WO_3_/TiO_2_ catalysts increased NO reduction activity. On the other hand, at a reaction temperature below 400 °C, only cobalt oxide substitution increased the NO reduction performance. Of all the partial substitution catalysts, the Co-VW catalyst demonstrated excellent NO reduction activity in SCR-NH_3_ and resulted in a low yield of N_2_O. The Co-VW supported on TiO_2_ catalyst displayed over 90% NO conversion at 350 to 495 °C reaction temperature under a high gas hourly space velocity (GHSV) of 300,000 h^−1^. In terms of inhibition effect of H_2_O and SO_2_ on the SCR activity, the substitution of cobalt into the VW/TiO_2_ catalyst enhanced the H_2_O and SO_2_ resistance significantly. From the characterisation studies, the active Co and V species and W promoter are observed to be well dispersed on the TiO_2_ support, after partial substitution of the catalyst. An increase in the amount of surface absorbed oxygen, and Brønsted and Lewis acid sites was observed when Co was introduced into the VW/TiO_2_ catalyst. Both the Brønsted and Lewis acid sites were involved in the SCR-NH_3_ of NO reaction. The activity of SCR-NH_3_ within the reaction temperature range was improved, as there was an increment in the number of Lewis and Brønsted acid sites, which is attributed to the Co substitution of V in the catalyst. Hence, unique bimetallic and ternary mixed oxide catalyst has the promising potential as a catalyst for controlling the emission of NO*_x_* from diesel engines.

Generally, low-temperature SCR reactions usually implement manganese oxides (MnO*_x_*)-based catalysts, as they show different types of labile oxygens, which are important for completing the NO catalytic cycle [58]. MnO*_x_*/TiO_2_ [132–133] and Mn deposited over carbon-ceramic cellular monolith were reported to be active at low SCR temperature in the presence of oxygen for the successful removal of NO with NH_3_ [134]. Ceria (CeO_2_), on the other hand, is known to be inexpensive and non-toxic. It is potentially advantageous compared to many catalytic applications since it has two stable oxidation states, Ce^4+^ and Ce^3+^. The redox shift that occurs between Ce^4+^ and Ce^3+^ could increase the ease of storing and release of oxygen in ceria. In addition, ceria could also increase the catalyst activity for the SCR-NH_3_ of NO by promoting the oxidisation of NO to NO_2_ [135]. Therefore, the mixed oxide Mn–Ce catalyst is considered to be one of the potential catalysts for high NO reduction activity at low SCR-NH_3_ temperature. Recently, Carja et al. [136] developed a Mn–Ce/ZSM-5 catalyst in aqueous phase. They reported that 75% to nearly 100% of NO was converted within a 240 to 500 °C temperature window. Qi et al. [137] also studied the Mn–Ce mixed oxide catalyst, which achived 100% NO conversion at a reaction temperature of 150 °C. Recently, Wu et al. [58] prepared a series of cerium-modified MnO*_x_* supported onto TiO_2_ using the sol–gel method. The main objective of the study was to determine the possible catalysts that would result in higher catalytic activities at low operating temperature (<100 °C). It was found that Ce–MnO*_x_*/TiO_2_ catalysts demonstrated a high level of NO activity at low SCR-NH_3_ temperature. Of all the cerium-modified MnO*_x_* catalysts, Ce(0.07)MnTi showed a high (80%) NO conversion at low (80 °C) SCR-NH_3_ temperature with a GHSV of 40,000 h^−1^. In terms of characterisation studies, the pore volume and BET surface area in the Ce(0.07)MnTi catalyst is 50% larger than that of the Ce(0)MnTi catalyst. The XPS results confirm that by introducing the Ce into the catalyst, the chemisorbed oxygen concentration on the catalyst surface could doubly increase. Besides that, the TPR results indicate that the activity of MnO*_x_*/TiO_2_ could be enhanced, the catalyst oxygen storage improved, and facilitation of oxygen mobility over the catalysts achieved, when Ce is present together with MnO*_x_*/TiO_2_. NH_3_-TPD justifies that the cerium-modified MnO*_x_*/TiO_2_ catalyst provides better NH_3_ chemisorption ability, which improves the SCR activity.

As mentioned previously, the existence of ceria helps in the process of oxidising NO to NO_2_, thereby increasing the NO reduction activity [58,138–139]. Unfortunately, pure ceria usually tends to lose its oxygen storage capacity (OSC), ending up in catalyst deactivation, as it is known to be poorly thermostable and undergoes rapid sintering at high reaction temperatures [140–141]. Hence, numerous efforts have been made to address this problem by incorporating another type of metal oxide into the ceria lattice. This would facilitate the formation of mixed oxides or solid solutions [141–143]. Moreover, the catalytic performance of the two mixed oxides will be improved, and a new stable compound with different physiochemical properties and catalytic behaviour can be obtained [140]. Xu et al. [144] developed a CeO_2_–TiO_2_ mixed oxide catalyst for the SCR of NO with NH_3_ in the presence of oxygen using the impregnation method. It was found that the catalyst was able to convert only up to 92% of NO within a 275 to 400 °C working temperature. Gao et al. [37], on the other hand, applied the sol–gel method to prepare the CeO_2_–TiO_2_ catalysts for SCR-NH_3_ of NO. The main focus of the study was to enhance the catalyst performance by achieving better dispersion of ceria onto the TiO_2_ support. From the activity results, it could be summarised that the CeO_2_–TiO_2_ catalyst prepared by the sol–gel method was highly active in the presence of oxygen. The CeO_2_–TiO_2_ catalyst with a 0.6 mass ratio was found to be the most optimum metal loading value that would result in a high conversion of NO. About 93.4 to 98.6% of NO was converted at a reaction temperature of 250–450 °C over the Ce(0.6)Ti catalyst. The remarkable performance portrayed by this catalyst is attributed to the strong interaction between Ce and Ti component. Besides that, the high concentration level of amorphous Ce on the catalyst surface as well as the increment in the chemisorbed oxygen or/and weakly bonded species, which is a result of the existence of Ce^3+^ after the introduction of Ce, are also some of the main factors that contribute towards the high catalytic activity.

#### Al_2_O_3_ as a catalyst support

Nowadays, gamma alumina (γ-Al_2_O_3_) has also been used as one of the main supports for SCR catalysts. This material has good physiochemical properties such as high specific area, high temperature resistance to inertia in both acid and alkali media, as well as excellent dispersion of the active component [60]. The γ-Al_2_O_3_ support is highly suitable for the SCR reaction since SCR is closely associated with the acidity of the catalyst, whereas the γ-Al_2_O_3_ surface occupied with both Brønsted and Lewis acid sites can easily absorb alkaline gas [145–146]. Recently, numerous types of Ce-based catalysts have been reported, such as CeO_2_/Al_2_O_3_ [147], MnO*_x_*–CeO*_y_*/γ-Al_2_O_3_ [148], and copper-modified Al–Ce-PILC [149]. Unfortunately, some of the Ce-based catalyst supported on Al_2_O_3_ have poor resistance against sulphur poisoning, which limits their application, especially in marine diesel engines [60]. Recently, many studies have implemented MoO_3_ as a second metal in the catalyst. MoO_3_ has a high compatibility with SO_2_, excellent resistance to arsenic poisoning, promotes the adsorption of ammonia, and has a strong solid acid content that enhances the acidity of the catalyst [150–151]. Liu et al. [152] found that high NO conversion was achieved at a broad temperature range of 300 to 450 °C, when 5% of MoO_3_ was loaded onto the CeO_2_/TiO_2_ catalyst. Lietti et al. [153] reported that the NO conversion improved as Mo was added onto the V_2_O_5_/TiO_2_ catalyst. Wang et al. [154] suggested that a high NO conversion could be obtained when MoO_3_ is impregnated with the HZSM-5 zeolite catalyst. Hence, Yan et al. [60] worked on developing a series of CeO_2_/Al_2_O_3_ and CeMo*_x_*O*_y_*/Al_2_O_3_ catalysts using an extrusion method in order to study the best catalytic performance of NO*_x_* activity. It was found that the molybdenum additives (CeMo*_x_*O*_y_*/Al_2_O_3_) portrayed more excellent catalytic activity than the CeO_2_/Al_2_O_3_ catalyst. About 95% of NO was converted over 250 to 430 °C working temperature under a GHSV of 7200 h^−1^. The best calcination temperature for CeMo*_x_*O*_y_*/Al_2_O_3_ catalyst was at 550 °C. The catalyst also demonstrated high resistance towards H_2_O and SO_2_ poisoning. The following reasons are some of the best features that reflect the promotional effect of molybdenum additives on the SCR-NH_3_ catalytic performance of NO*_x_*: (a) the amount of surface acidity increases when molybdenum is used as additive in the catalyst; (b) the specific surface area increases as molybdenum is applied as additive; (c) a higher proportion of Ce^3+^/(Ce^4+^ + Ce^3+^) and O_α_/(O + O_β_) on the catalyst surface is produced, which leads to an increment in catalyst activity. Therefore, the CeMo*_x_*O*_y_*/Al_2_O_3_ catalyst can also be applied to control the emission of NO*_x_* in the SCR-NH_3_ of NO reaction. Table 5 summarises the reaction conditions and NO activity of non-carbon-based catalyst supports.

**Table 5:** Summary on the reaction conditions and NO activity for non-carbon supported catalysts.

Catalyst	Preparation method									
	Activation conditions									
	Reaction conditions							Best NO removal		Refs.
	NO (ppm)	NH_3_ (ppm)	O_2_ (%)	SO_2_ (ppm)	Balance (gas)	Flow rate (mL/min)	GSHV (h^−1^)	Conversion (%)	Temperature (°C)	

CuCe/ZSM-5	conventional ion-exchange									
	calcination: 600 °C/4 h									
	1000	1000	10	–	N_2_	–	15,000	95	148–427	[63]
Mn/CeTi Mn/CeSn	inverse co-precipitation									
	calcination: 550 °C/5 h									
	500	500	5	100	N_2_	–	60,000	>90	175–300	[155]
M*_x_*O*_y_*–V_2_O_5_–WO_3_/TiO_2_	impregnation									
	calcination: 550 °C/3 h (Air)									
	1000	1000	10	–	N_2_	2.51	60,000	>90	350–495	[59]
Ce–MnO*_x_*/TiO_2_	sol-gel									
	calcination: 500 °C/6 h (Air)									
	1000	1000	3	–	N_2_	–	40,000	84	80	[58]
CeMo*_x_*O*_y_*/Al_2_O_3_	extrusion									
	calcination: 550 °C/2 h (Air)									
	930	930	10	475	N_2_	1200	7,200	>95	250–430	[60]
CeO_2_–TiO_2_	sol–gel									
	calcination: 500 °C/5 h (Air)									
	1000	1000	3	200	N_2_	–	–	98.6	300–400	[124]
Mn/ZSM-5-t	precipitation									
	calcination: 200-700 °C									
	600	600	4.5	–	N_2_	300	36,000	≈100	150–390	[51]
γ-Fe_2_O_3_ α-Fe_2_O_3_	co-precipitation									
	calcination: 250 °C/2 h (γ-Fe_2_O_3_), 500 °C/5 h (α-Fe_2_O_3_)									
	500	500	3	–	N_2_	300	47,000	90	200–300	[156]
Ce–Sn–Ti	inverse co-precipitation									
	calcination: 500 °C/6 h									
	800	800	5	200	N_2_	500	50,000	>90	280–400	[157]
Fe–Mn/TiO_2_	incipient wetness impregnation									
	calcination: 500 °C/6 h (Air)									
	1000	1000	2	100	N_2_	100	30,000	≈100	120	[125]

## Conclusion

Carbon-based and non-carbon-based materials as catalyst supports for the selective catalytic reduction (SCR)-NH_3_ of NO have been comprehensively studied over the past few decades. The mechanism of SCR is acknowledged as a complex process that follows both the Langmuir–Hinshelwood and the Eley–Rideal mechanisms. The standard SCR usually has a lower reaction rate than the fast SCR, where the high formation of NO_2_ in the latter contributes more efficiently as an oxidising agent in the redox reaction. The modification of catalyst supports, focusing on their physiochemical properties, was thoroughly reviewed. The preparation method is one of the most crucial factors that affect the nature and dispersion of metal loading onto the catalyst surface. Several methodological procedures such as sol–gel, impregnation, extrusion, reflux, and pyridine-thermal are among the more frequently used methods for achieving high catalytic performance. In this review, the performance of carbon-based materials as catalyst supports, including carbon nanotubes, activated carbon, activated carbon fibre, and graphene on the SCR-NH_3_ of NO activity was discussed in detailed. In comparison, non-carbon-based catalyst supports such as ZSM-5, TiO_2_, and Al_2_O_3_ were also reviewed. In summary, carbon nanotube-based catalyst supports were found to yield the best NO conversion, even at low operating temperatures, out of all of the catalyst supports studied. Pure CNTs yield low NO conversion and have poor resistance to H_2_O and SO_2_. However, catalyst modification with other metal oxides (CeO*_x_*, MnO*_x_*, FeO*_x_*, CuO*_x_*) has been proven to be promising in attaining novel catalysts with high NO reduction activity. Even though many attempts have previously been made, scientists need to conduct more in-depth and far-reaching studies in order to achieve the best NO conversion for actual industrial and power emission applications. Moreover, other suggestions of a technical nature are also important to produce high-end research projects. Further recommendations that can be considered are:

1. Implementing the microwave irradiation technique for activation of CNT-based catalyst supports.
2. Performing a research study on simultaneous removal of SO_2_ and NO using the best CNT-based supported catalyst.
3. Fundamental study on the electronic interaction between CNTs and metal oxides in the SCR-NH_3_ system using a density functional theory (DFT) approach.

## References

[1] Wang M, Liu H, Huang Z-H, Kang F (2014). Chem Eng J.

[2] Sumathi S, Bhatia S, Lee K T, Mohamed A R (2010). J Hazard Mater.

[3] Liu C, Shi J-W, Gao C, Niu C (2016). Appl Catal, A.

[4] Valdes S, Marban G, Fuertes A B (2003). Appl Catal, B: Environ.

[5] Mrad R, Aissat A, Cousin R, Courcot D, Siffert S (2015). Appl Catal, A.

[6] You X, Sheng Z, Yu D, Yang L, Xiao X, Wang S (2017). Appl Surf Sci.

[7] Pan S, Luo H, Li L, Wer Z, Huang B (2013). J Mol Catal A: Chem.

[8] Shan W, Song H (2015). Catal Sci Technol.

[9] Li J, Chang H, Ma L, Hao J, Yang R T (2011). Catal Today.

[10] Cao F, Su S, Xiang J, Wang P, Hu S, Sun L, Zhang A (2015). Fuel.

[11] Li Y, Wan Y, Li Y, Zhan S, Guan Q, Tian Y (2016). ACS Appl Mater Interfaces.

[12] Rahmaninejad F, Gavaskar V S, Abbasian J (2012). Appl Catal, B: Environ.

[13] Shen B, Wang Y, Wang F, Liu T (2014). Chem Eng J.

[14] Macleod N, Lambert R M (2002). Appl Catal, B: Environ.

[15] Pereira M V L, Nicolle A, Berthout D (2015). Catal Today.

[16] Jin R, Liu Y, Wu Z, Wang H, Gu T (2010). Chemosphere.

[17] Lázaro M J, Ascaso S, Pérez-Rodríguez S, Calderón J C, Gálvez M E, Nieto M J, Moliner R, Boyano A, Sebastián D, Alegre C (2015). C R Chim.

[18] Chuang K-H, Lu C-Y, Wey M-Y, Huang Y-N (2011). Appl Catal, A.

[19] Cha W, Ehrman S H, Jurng J (2016). J Environ Chem Eng.

[20] Nova I, Ciardelli C, Tronconi E, Chatterjee D, Bandl-Konrad B (2006). Catal Today.

[21] Liu Z, Yi Y, Zhang S, Zhu T, Zhu J, Wang J (2013). Catal Today.

[22] Liu Z, Su H, Chen B, Li J, Woo S I (2016). Chem Eng J.

[23] Xu H, Wang Y, Cao Y, Fang Z, Lin T, Gong M, Chen Y (2014). Chem Eng J.

[24] Wei Z S, Zeng G H, Xie Z R, Ma C Y, Liu X H, Sun J L, Liu L H (2011). Fuel.

[25] Shi Y, Chen S, Sun H, Shu Y, Quan X (2013). Catal Commun.

[26] Chen L, Li J, Ge M, Ma L, Chang H (2011). Chin J Catal.

[27] Liu F, Asakura K, He H, Liu Y, Shan W, Shi X, Zhang C (2011). Catal Today.

[28] Zhang D, Zhang L, Fang C, Gao R, Qian Y, Shi L, Zhang J (2013). RSC Adv.

[29] Sohrabi S, Akhlaghian F (2016). J Nanostruct Chem.

[30] Chong S, Zhang G, Zhang N, Liu J, Zhu Y, Huang T, Fang S (2016). Ultrason Sonochem.

[31] Zhang H, Chu W, Zou C, Huang Z, Ye Z, Zhu L (2011). Catal Lett.

[32] Yang Y, Chiang K, Burke N (2011). Catal Today.

[33] Dong Y, Ren X, Wang M, He Q, Chang L, Bao W (2013). J Energy Chem.

[34] Jeong B, Ye B, Kim E-S, Kim H-D (2017). Catal Commun.

[35] Qi K, Xie J, Fang D, Li F, He F (2017). Chin J Catal.

[36] Nam K B, Kwon D W, Hong S C (2017). Appl Catal, A.

[37] Gao X, Jiang Y, Zhong Y, Luo Z, Cen K (2010). J Hazard Mater.

[38] Yeoh W-M, Lee K-Y, Chai S-P, Lee K-T, Mohamed A R (2013). J Phys Chem Solids.

[39] Xiong Z-b, Peng B, Zhou F, Wu C, Lu W, Jin J, Ding S-f (2017). Powder Technol.

[40] Yao X, Chen L, Kong T, Ding S, Luo Q, Yang F (2017). Chin J Catal.

[41] Zeng Y, Wang T, Zhang S, Wang Y, Zhong Q (2017). Appl Surf Sci.

[42] Zhu L, Zeng Y, Zhang S, Deng J, Zhong Q (2017). J Environ Sci.

[43] Noor Azeerah A, Haliza A A, Zulina A M (2012). Co-precipitation technology for preparation of solid catalyst in oleochemical processes.

[44] Zhang Y, Zheng Y, Wang X, Lu X (2015). Catal Commun.

[45] Zhang F, Zhang S, Guan N, Schreier E, Richter M, Eckelt R, Fricke R (2007). Appl Catal, B: Environ.

[46] Sun P, Guo R-t, Liu S-m, Wang S-x, Pan W-g, Li M-y (2017). Appl Catal, A.

[47] Qi G, Yang R T (2003). Appl Catal, B: Environ.

[48] Debecker D P, Hulea V, Mutin P H (2013). Appl Catal, A.

[49] Fan M-S, Abdullah A Z, Bhatia S (2009). ChemCatChem.

[50] Su Y, Fan B, Wang L, Liu Y, Huang B, Fu M, Chen L, Ye D (2013). Catal Today.

[51] Lou X, Liu P, Li J, Li Z, He K (2014). Appl Surf Sci.

[52] Bai S, Li H, Wang L, Guan Y, Jiang S (2014). Catal Lett.

[53] Fang C, Zhang D, Shi L, Gao R, Li H, Ye L, Zhang J (2013). Catal Sci Technol.

[54] Bai S, Jiang S, Li H, Guan Y (2015). Chin J Chem Eng.

[55] Wang X, Zheng Y, Xu Z, Liu X, Zhang Y (2014). Catal Commun.

[56] Ma Z, Yang H, Li Q, Zheng J, Zhang X (2012). Appl Catal, A.

[57] Wang X, Zheng Y, Lin J (2013). Catal Commun.

[58] Wu Z, Jin R, Liu Y, Wang H (2008). Catal Commun.

[59] Zhang Q-m, Song C-l, Lv G, Bin F, Pang H-t, Song J-o (2015). J Ind Eng Chem.

[60] Yan W, Shen Y, Zhu S, Jin Q, Liu Y, Li X (2016). Catal Lett.

[61] Wu C, Sun X, Shen B, Williams P T (2014). J Energy Inst.

[62] Dou B, Lv G, Wang C, Hao Q, Hui K (2015). Chem Eng J.

[63] Muradov N, Smith F, T-Raissi A (2005). Catal Today.

[64] Rodgiguez-Reinoso F (1998). Carbon.

[65] Santos A, Yustos P, Quintanilla A, García-Ochoa F (2005). Top Catal.

[66] Auer E, Freund A, Pietsch J, Tacke T (1998). Appl Catal, A.

[67] Stüber F, Font J, Fortuny A, Bengoa C, Eftaxiaz A, Fabregat A (2005). Top Catal.

[68] Li X, Dong Z, Dou J, Yu J, Tahmasebi A (2016). Fuel Process Technol.

[69] Samojeden B, Grzybek T (2016). Energy.

[70] Zhang D, Zhang L, Shi L, Fang C, Li H, Gao R, Huang L, Zhang J (2013). Nanoscale.

[71] Planeix J M, Coustel N, Coq B, Brotons V, Kumbhar P S, Dutarte R, Geneste P, Bernier P, Ajayan P M (1994). J Am Chem Soc.

[72] Santillan-Jimenez E, Miljković-Kocić V, Crocker M, Wilson K (2011). Appl Catal, B: Environ.

[73] Santillan-Jimenez E, Crocker M, Bueno-López A, Salinas-Martínez de Lecea C (2011). Ind Eng Chem Res.

[74] Luo J Z, Gao L Z, Leung Y L, Au C T (2000). Catal Lett.

[75] Long R Q, Yang R T (2001). Ind Eng Chem Res.

[76] Huang Z, Zhu Z, Liu Z, Liu Q (2003). J Catal.

[77] Phil H H, Reddy M P, Kumar P A, Ju L K, Hyo J S (2008). Appl Catal, B: Environ.

[78] Li Q, Yang H, Ma Z, Zhang X (2012). Catal Commun.

[79] Zhu Z, Niu H, Liu Z, Liu S (2000). J Catal.

[80] Shen Y, Zhu S (2012). Catal Sci Technol.

[81] Jang S, Li J, Wang C, Chen J, Ma L, Chang H, Chen L, Peng Y, Yan N (2012). Appl Catal, B: Environ.

[82] Wang L, Huang B, Su Y, Zhaou G, Wang K, Luo H, Ye D (2012). Chem Eng J.

[83] Wang X, Zheng Y, Xu Z, Liu Y, Wang X (2014). Catal Sci Technol.

[84] Tang X, Hao J, Xu W, Li J (2007). Catal Commun.

[85] Kijlstra W S, Brands D S, Smit H I, Poels E K, Bliek A (1997). J Catal.

[86] Cao F, Zhong K, Gao A, Chen C, Li Q, Chen Q (2007). J Phys Chem B.

[87] Chen W, Pan X, Willinger M-G, Su D S, Bao X (2006). J Am Chem Soc.

[88] Chen W, Pan X, Bao X (2007). J Am Chem Soc.

[89] Zhu Z, Liu Z, Niu H, Liu S, Hu T, Xie Y (2001). J Catal.

[90] Chen J P, Yang R T (1990). J Catal.

[91] Chen J P, Yang R T (1993). J Catal.

[92] Huang B, Huang R, Jin D, Ye D (2007). Catal Today.

[93] Buitrago R, Ruiz-Martínez J, Serrano-Ruiz J C, Rodríguez-Reinoso F, Sepúlved-Escribano A (2012). J Colloid Interface Sci.

[94] Zhang S, Li Q, Zhong H (2012). Appl Catal, A: Gen.

[95] Tsen H-H, Lub C-Y, Chang F-Y, Weyd M-Y, Chang H-T (2011). Chem Eng J.

[96] Zhu L, Huang B, Wang W, Wei Z, Ye D (2011). Catal Commun.

[97] Mochida I, Koraia Y, Shirahama M, Kawano S, Hada T, Seo Y, Yoshikawa M, Yasutake A (2000). Carbon.

[98] Yoon K S, Ryu S K (2010). Korean J Chem Eng.

[99] Sousa J P S, Pereira M F R, Figueiredo J L (2013). Fuel Process Technol.

[100] Klinik J, Samojeden B, Grzybek T, Suprun W, Papp H, Gläser R (2011). Catal Today.

[101] Boyano A, Iritia M C, Malpartida I, Larrubia M A, Alemany L J, Moliner R, Lázaro M J (2008). Catal Today.

[102] Muñiz J, Marbán G, Fuertes A B (1999). Appl Catal, B: Environ.

[103] Lázaro M L, Boyano A, Gálvez M E, Izquierdo M T, Garcia-Bordejé E, Ruiz C, Juan R, Moliner R (2008). Catal Today.

[104] Gálvez M E, Lázaro M J, Moliner R (2005). Catal Today.

[105] Huang M-C, Teng H (2003). Carbon.

[106] Gao X, Liu S, Zhang Y, Du X, Luo Z, Cen K (2011). Catal Today.

[107] Yang N, Yu J-l, Dou J-x, Tahmasebi A, Song H, Moghtaderi B, Lucas J, Wall T (2016). Fuel Process Technol.

[108] Lua P, Li C, Zenga G, Heb L, Penga D, Cuia H, Li S, Zhai Y (2010). Appl Catal, B: Environ.

[109] Athappan A, Sattler M L, Sethupathi S (2015). J Environ Chem Eng.

[110] Castegnaro M V, Killian A S, Baibich I M, Alves M C M, Morais J (2013). Langmuir.

[111] Rathrore R S, Srivastava D K, Agarwal A K, Verma N (2010). J Hazard Mater.

[112] Talukdar P, Bhaduri B, Verma N (2014). Ind Eng Chem Res.

[113] Lu X, Song C, Chang C-C, Yeng Y, Tong Z, Tang X (2014). Ind Eng Chem Res.

[114] Guo Z, Wang M, Huang Z-H, Kang F (2015). Carbon.

[115] Xiao X, Sheng Z, Yang L, Dong F (2016). Catal Sci Technol.

[116] Lu X, Song S, Jia C, Tong Z, Tang X, Teng Y (2015). Chem Eng J.

[117] Wang M-X, Huang Z-H, Shen K, Kang F, Liang K (2013). Catal Today.

[118] Su W, Lu X, Jia S, Wang J, Ma H, Xing Y (2015). Catal Lett.

[119] Jiao J-Z, Li S-H, Huang B-C (2015). Acta Phys-Chim Sin.

[120] Fidalgo B, Menéndez J A (2011). Chin J Catal.

[121] Greenhalgh B, Fee M, Dobri A, Moir J, Burich R, Charland J-P, Stanciulescu M (2010). J Mol Catal A: Chem.

[122] Liu F, He H, Ding Y, Zhang C (2009). Appl Catal, B: Environ.

[123] Picasso G, Gutiérrez M, Pina M P, Herguido J (2007). Chem Eng J.

[124] Katta L, Sudarsanam P, Thrimurthulu G, Reddy B M (2010). Appl Catal, B: Environ.

[125] Laguna O H, Sarria F R, Centeno M A, Odriozola J A (2010). J Catal.

[126] Pang L, Fan C, Shao L, Song K, Yi J, Cai X, Wang J, Kang M, Li T (2014). Chem Eng J.

[127] Thirupathi B, Smirniotis P G (2011). Catal Lett.

[128] Shan W, Liu F, He H, Shi X, Zhang C (2012). Appl Catal, B: Environ.

[129] Ma Z, Weng D, Wu X, Si Z, Wang B (2012). Catal Commun.

[130] Shi A, Wang X, Yu T, Shen M (2011). Appl Catal, B: Environ.

[131] Lee D W, Yoo B R (2014). J Ind Eng Chem.

[132] Wu Z, Jiang B, Liu Y, Zhao W, Guan B (2007). J Hazard Mater.

[133] Ettireddy P R, Ettireddy N, Mamedov S, Boolchand P, Smirniotis P G (2007). Appl Catal, B: Environ.

[134] Valdés-Solís T, Marbán G, Fuertes A B (2001). Catal Today.

[135] Qi G, Yang R T, Chang R (2004). Appl Catal, B: Environ.

[136] Carja G, Kameshima Y, Okada L, Madhusoodana C D (2007). Appl Catal, B: Environ.

[137] Qi G, Yang R T (2003). J Catal.

[138] Long R Q, Yang R T (2000). Appl Catal, B: Environ.

[139] Long R Q, Tang R T (1999). J Am Chem Soc.

[140] Reddy N M, Khan A, Yamada Y, Kobayashi T, Loridant S, Volta J-C (2003). J Phys Chem B.

[141] Luo M, Chen J, Chen L, Lu L, Feng Z, Li C (2001). Chem Mater.

[142] Reddy B M, Laskmanan P, Khan A (2004). J Phys Chem B.

[143] Wu Z, Jiang B, Liu Y, Wang H, Jin R (2007). Environ Sci Technol.

[144] Xu W, Yu Y, Zhang C, He H (2008). Catal Commun.

[145] Yan S, Wang X, Wang W, Liu Z, Niu J (2012). J Nat Gas Chem.

[146] Tonetto G M, Damiani D E (2003). J Mol Catal A: Chem.

[147] Shen Y, Zhu S, Qiu T, Shen S (2009). Catal Commun.

[148] Qu L, Li C, Zeng G, Zhang M, Fu M, Ma J, Zhan F, Luo D (2014). Chem Eng J.

[149] Lin Q, Hao J, Li J, Ma Z, Lin W (2007). Catal Today.

[150] Djerad S, Crocoll M, Kureti S, Tifouti L, Weisweiler W (2006). Catal Today.

[151] Djerad S, Tifouti L, Crocoll M, Weisweiler W (2004). J Mol Catal A: Chem.

[152] Liu Z, Zhang S, Li J, Ma L (2014). Appl Catal, B: Environ.

[153] Lietti L, Nova I, Ramis G, Dall’Acqua L, Busca G, Giamello E, Forzatti P, Bregani F (1999). J Catal.

[154] Wang X, Yu S, Yang H, Zhang S (2007). Appl Catal, B: Environ.

[155] Xiong Y, Tang C, Tao X, Zhang L, Li L, Wang X, Deng Y, Gao F, Dong L (2015). Appl Catal, A.

[156] Liu C, Yang S, Ma L, Peng Y, Hamidreza A, Chang H, Li J (2013). Catal Lett.

[157] Yu M, Li C, Zeng G, Zhou Y, Zhang X, Xie Y (2015). Appl Surf Sci.

